# Group Dominance, System Justification, and Hostile Classism: The Ideological Roots of the Perceived Socioeconomic Humanity Gap That Upholds the Income Gap

**DOI:** 10.5334/irsp.753

**Published:** 2023-06-08

**Authors:** Mario Sainz, Gloria Jiménez-Moya

**Affiliations:** 1 Departamento de Psicología Social y de las Organizaciones, Facultad de Psicología, Universidad Nacional de Educación a Distancia, Spain; 2 Escuela de Psicología, Pontificia Universidad Católica de Chile, Santiago, Chile

**Keywords:** ideology, social dominance, system justification, classism, dehumanisation, poverty, wealth, attributions, redistribution

## Abstract

Perceiving low-socioeconomic status (low-SES) groups as less human than high-SES groups contributes to justifying socioeconomic inequality. Despite this issue’s relevance, previous research has not acknowledged the possible causes of this perceived humanity gap (differences in humanity between SES groups). In this project, we focus on analysing the possible influence of hierarchy-enhancing ideological variables on this gap. To do so, in a first correlational study (*N* = 765), we analyse the extent to which certain ideological variables predict the perceived humanity gap between low- and high-SES groups. Our results indicate that group dominance, system justification, and hostile classism are highly predictive of the humanity gap. In a second correlational study (*N* = 521) we found that the perceived humanity gap, the tendency to blame low-SES groups and praise high-SES groups for their economic standings, sequentially mediated the relationship among social dominance, system justification, and hostile classism with the support of social change policies. Finally, we manipulated each ideological variable in three equivalent studies (*N* = 631) to test its influence on the previous pattern of mediational results. The results confirmed the ideological variables’ antecedent roles in the mediation analysis. Finally, we discuss the role of the ideological hierarchy variables in the maintenance of socioeconomic differences through (de)humanisation.

The consequences of attributing more humanity to high-socioeconomic status (high-SES) groups than to low-SES groups are severe; it contributes to maintaining economic inequality and justifying socioeconomic differences (Sainz, Loughnan et al., 2020; Sainz, Martínez, Rodríguez-Bailón & Moya, 2019). Because of this issue’s social relevance, it is important to determine the factors that trigger this perceived humanity gap between socioeconomic groups. Regarding this matter, previous research on the socioeconomic realms is scarce. Therefore, a detailed analysis of the variables that can explain the perceived humanity gap that upholds socioeconomic differences is necessary. In the present project, we focus on the hierarchy-enhancing ideological variables, which are tidily related to not only the tendency to dehumanise others (Haslam & Loughnan, 2014; Vaes et al., 2012) but also the legitimisation of economic inequality (Rodríguez-Bailón et al., 2017).

## Hierarchy-Enhancing Ideologies and the Justification of the Socioeconomic Gap

There is a vast amount of research analysing individual ideological standing’s role as a trigger of several psychosocial processes, such as intergroup conflicts or negative attitudes towards others (Hodson & Dhont, 2015). In this regard, previous research has identified a variety of hierarchy-enhancing ideologies that provide individuals with a set of moral and intellectual reasons that lead them to assign social value to other individuals or groups based on their (dis)advantaged position (Sidanius & Pratto, 1999). This is the case of social dominance orientation (SDO), which reflects personal preferences for hierarchy-based interactions (vs. equal relations) and the belief that advantaged groups should dominate less powerful groups (Pratto et al., 1994); or system justification (SJ) beliefs, which imply a set of legitimisation myths about how status differences reflect fair and merited outcomes (Jost & Banaji, 1994). Additionally, these two sets of beliefs usually align with political conservative or right-wing positions that defend traditional values in opposition to social change (Jost et al., 2003). Further, recent work has also analysed the role of tolerance to inequality (Wiwad et al., 2019) that captures individuals’ preference and tolerance for the existing socioeconomic gap within societies; and the ambivalent attitudes towards the poor, ranging from the more hostile (i.e., the explicit derogation of poor people and groups) to the more paternalistic (i.e., the benevolent but patronising perception of poor people and groups) beliefs (Jordan et al., 2020) as hierarchy enhancing factors.

All of these variables contribute to justifying economic inequality and opposing egalitarian policies (Cervone et al., 2020; Jordan et al., 2020; Jost et al., 2003; Piff et al., 2020) by capturing related but distinct facets of how people understand differences among socioeconomic groups. On this matter, while SDO seems to capture people’s desires and preference for hierarchies, SJ beliefs refer to people’s understanding on how merit is ascribed as a function of personal effort, tolerance to inequality captures the extent to which people consider that inequality is a socially shared problem, and classism describe the attitudes towards those who have less. The distinctions among these factors are important elements to take in consideration, as the combination of different levels of each variable create specific set of attitudes that lead individuals to oppose social change, based on distinct reasons. Therefore, focusing on different aspects of people’s understanding of inequality could provide a more detailed picture of how each variable contribute to justify the socioeconomic gap.

In short, despite the specific contribution of each factor, it is clear that the adherence to this set of hierarchy-enhancing ideologies shapes the understanding of society as a whole by amplifying the accomplishments of advantaged (vs. disadvantaged) groups (Kteily et al., 2019) and legitimising socioeconomic differences (Rodríguez-Bailón et al., 2017). Therefore, it is important to address the role of all of these variables as they contribute to perceiving low-SES groups as lacking high-SES groups’ traits and virtues, which justifies the unequal distribution of resources, goods, and services within societies and promotes the maintenance of the status quo.

## Attribution of Humanity to Low-SES and Hig-HSES Groups

The (de)humanisation of others has been identified as a key process in intergroup relationships (for reviews, see Haslam & Loughnan, 2014; Haslam & Stratemeyer, 2016; Kteily & Landry, 2022; Vaes et al., 2012). The tendency to perceive others as less than human has also been found to be relevant in the socioeconomic domain. Compared to high-SES groups, low-SES groups are usually considered to be less evolved and to have less human uniqueness traits, such as rationality and culture (i.e., animalistic dehumanisation; Loughnan et al., 2014; Sainz, Martínez, Moya & Rodríguez-Bailón, 2019). Furthermore, high-SES groups are perceived as more evolved in terms of human uniqueness traits but lacking in other human nature traits, such as emotionality and interpersonal warmth (i.e., mechanistic dehumanisation; Sainz, Martínez, Moya & Rodríguez-Bailón, 2019).

This tendency to differently and complementarily attribute humanity as a function of SES has severe consequences on the attributional process of poverty and wealth, as well as on the tendency to support social change policies. First, research shows that animalistically dehumanising those at the bottom of society leads people to blame this group for their plight (Sainz, Loughnan et al., 2020; Sainz, Martínez et al., 2020). This implies that people make more internal attributions (e.g., believing poverty is caused by the poor’s lack of motivation or abilities) rather than external attributions about poverty (e.g., considering that poverty is caused by discrimination or economic recessions) when they dehumanise low-SES groups. Second, humanising high-SES groups (in terms of higher ascription of human nature traits) creates the perception that high-SES groups deserve their standing (Sainz, Martínez, Rodríguez-Bailón & Moya, 2019). This implies that when humanising high-SES groups, individuals will make more internal attributions (e.g., believing wealth is the result of rich people’s personal effort and motivation) than external ones (e.g., considering that wealth is caused by inheriting relatives’ resources or by political pull) about high-SES groups’ wealth. Furthermore, both processes favour the rejection of social change policies. For instance, dehumanising low-SES groups and humanising high-SES groups has been independently found to lead to rejection of the implementation of redistribution policies, progressive taxation systems, and welfare policies aimed at helping those in need (Sainz, Loughnan et al., 2020; Sainz, Martínez et al., 2020; Sainz, Martínez, Rodríguez-Bailón & Moya, 2019).

Based on these previous findings, we acknowledge the importance of (de)humanising low- and high-SES groups as the cause of the attributional processes and support for measures that defy the status quo. So far, however, there is scarce research trying to analyse the variables that might explain the perceived humanity gap in the socioeconomic domain’s specific context.

## Overview

We have acknowledged certain ideologies’ role in the perpetration of dehumanisation of a variety of groups in intergroup conflicts (Banton et al., 2020; DeLuca-McLean & Castano, 2009; Esses et al., 2008; Hodson & Costello, 2007; Russo & Mosso, 2019; Trounson et al., 2015). Generally, individual adherence to these hierarchy-enhancing ideologies is associated with a greater tendency to dehumanise disadvantaged groups (e.g., immigrants, low-status groups) and with other similar outcomes (e.g., rejecting immigration policies, supporting punitive justice). Based on this previous literature, we aimed to test the effect of ideological variables (1) on the perceived humanity gap (in terms of human uniqueness traits) between low- and high-SES groups and (2) on social change policies, considering the mediating role of the humanity gap between socioeconomic groups and attributions of poverty and wealth in this association. Our contribution is threefold. First, we test the impact of the hierarchy-enhancing ideologies on the perceived humanity gap taking into account several ideologies that have been identified in previous research. Second, when addressing the perceived humanity gap, we specifically focus on the human uniqueness dimension (i.e., animalistic dehumanisation) captured by the blatant dehumanisation scale (Kteily et al., 2015). This focus was motivated by the understanding that the human uniqueness dimension is considered as a hierarchy-based dehumanisation form that is commonly applied to distinguish between disadvantaged (e.g., low-SES groups) and advantaged groups (e.g., high-SES groups) and, further, influence on the maintenance of the social hierarchies and the existing unequal status quo (Haslam, 2006; Haslam & Loughnan, 2014; Sainz, Martínez, Moya & Rodríguez-Bailón, 2019). Third, we accounted for the humanity of not only low-SES groups—as the vast majority of previous research—but also high-SES groups. This consideration was aimed at testing the effect of the humanity gap (on human uniqueness traits) on attributional processes regarding poverty and wealth as well as on different social change policies, such as providing help to the poor or increasing tax pressure on wealthy individuals and groups.

To achieve our goals, in a Pilot Study, we analysed the relationship between the ideological variables and the humanity gap on human uniqueness traits between low- and high-SES groups. In Study 1, we tested the more effect relevant ideological predictors, according to the Pilot Study (i.e., social dominance, SJ beliefs, and hostile classism) on support for social change policies, including the humanity gap and the attributions of poverty and wealth, as mediating variables in this relation. In Study 2, we ran three experimental studies indirectly manipulating each ideological variable (social dominance, SJ beliefs, and hostile classism) to test its causal effect on the previously identified pattern of results.

## Pilot Study

In this study, we aimed to explore the extent to which certain ideological variables predict the humanity gap between low- and high-SES groups. To do so, we selected a group of ideologies that have been explored in previous research: SDO, SJ, political orientation, tolerance to inequality, and ambivalent classism. We expected that higher (vs. lower) endorsement of these ideological variables would predict a larger humanity gap between low- and high-SES groups.^1^ The hypothesis’s preregistration can be found online: https://osf.io/vserq

## Method

### Participants and Procedure

As preregistered, to test our hypotheses, we used data already collected for a previous project.^2^ This data set contains 765 Mexican participants (314 women, 445 men, and 6 others; *M*_age_ = 26.01; *SD* = 7.37) recruited using Prolific Academic services (where participants were paid 1£ each). We conducted post hoc power analysis for a small–medium effect size (multiple regression, ten predictors, α = .05, *f*^2^ = .03) using G*Power analysis (Faul et al., 2009). The analysis indicated that we reached 93% Power, allowing us to confirm that the sample size was sufficiently large to test our hypothesis. The participants answered the following scales:

#### Ideological Orientation

To measure hierarchically enhanced ideologies that could uphold the economic gap, we included the following variables: (1) SDO (8 items and 2 subfactors: dominance orientation, e.g., ‘Some groups of people are simply inferior to other groups,’ α = .66 and antiegalitarianism, e.g., ‘Group equality should not be our ideal,’ α = .72; Ho et al. 2015); (2) SJ beliefs (seven items, e.g., ‘If people work hard, they almost always get what they deserve,’ α = .85; adapted version for Spanish speakers from Jaume et al., 2012); (3) political orientation (single item from *Extreme left* to *Extreme right*); (4) tolerance for economic inequality (five items, e.g., ‘Economic inequality is causing many of the world’s problems,’ α = .75; Wiwad et al., 2019), and (5) ambivalent classism (20 items, 3 subfactors: hostile classism, e.g., ‘Many poor people cannot be trusted to make important life decisions for themselves,’ α = .94; protective paternalism, e.g., ‘Charitable organisations should help poor people use their food stamps wisely,’ α = .89; and complementary class differentiation, e.g., ‘Poor people are often more humble than nonpoor people’; α = .82; Sainz et al., 2021). To run the analyses, we included each subfactor as a predictor of the perceived humanity gap, given that each of them captures different dimensions of the concepts. The participants answered using a scale ranging from 1 (*Completely disagree* and *Extreme left* for political orientation measure) to 7 (*Completely agree* and *Extreme right* for political orientation).

#### Humanity Gap

As in previous research that addressed dehumanization in the socioeconomic domain (Sainz, Martínez et al., 2020) participants rated the humanity of low-SES and poor groups (*r* = .909, *p* < .001), high-SES and rich groups (*r* = .880, *p* < .001), and other filler groups (e.g., politicians)—to mask the study’s purpose—using a slider from 0 (*least evolved/animal-like*) to 100 (*most evolved/human-like*; Ascent of Man Scale, Kteily et al., 2015). We computed an index of the humanity gap between low- and high-SES groups (high-SES/rich groups’ humanity scores minus low-SES/poor groups’ humanity scores).

Finally, participants reported their subjective SES (10-step MacArthur ladder from 1 *Low SES* to 10 *High SES*; Adler et al., 2001), their objective SES (monthly household income per person divided in deciles; Kraus & Keltner, 2009), and some demographic information (gender, age, native language, and nationality).

## Results and Discussion

First, we computed descriptive analysis and correlations between the variables included in the study (Table 1). The results show that, in general, ideological variables seem to be positively related to the humanity gap between low- and high-SES groups: Higher endorsement of ideological variables that justify inequality is related to a wider humanity gap between SES groups, with the exception of the non-significant correlation of one of the subfactors of ambivalent classism—namely, complementary class differentiation—with the perceived humanity gap. Furthermore, results highlight the previously identified differences in humanity attribution between high- (*M* = 65.85; *SD* = 21.81) and low-SES groups (*M* = 59.53; *SD* = 21.55; *t*(764) = 8.42; *p* < .001; *d* = .29). Second, we computed regression analysis to test our hypothesis. Results indicated that one of the factors of SDO, group dominance (not anti-egalitarianism), SJ beliefs, and hostile classism were the variables that positively predicted the humanity gap between low- and high-SES groups (Table 1).

**Table 1:** Descriptive Bivariate Correlations with Humanity Gap and Multiple Regression Analysis of Ideological Variables and Humanity Gap, Controlled for Participants’ Socioeconomic Status (Pilot Study).


	DESCRIPTIVE	CORRELATIONS	REGRESSION ON THE HUMANITY GAP	

			*F*_(10, 739)_ = 25.32**, *R*^2^ = .245	

	MEANS (*SDs*)	*r*	*β (SE)*	95% CI

Group dominance	2.75 (*1.24*)	.382**	3.34 (*.735*)**	[1.90; 4.79]

Anti-egalitarianism	2.47 (*1.14*)	.255**	–.74 (*.814*)	[–2.34; .86]

System justification	2.12 (*0.97*)	.212**	2.02 (*.724*)**	[.60; 3.44]

Political orientation	3.94 (*1.26*)	.363**	.90 (*.649*)	[–.37; 2.18]

Tolerance to inequality	3.73 (*1.14*)	.240**	–.57 (*.873*)	[–2.28; 1.14]

Hostile classism	2.74 (*1.34*)	.438**	4.22 (*.682*)**	[2.88; 5.56]

Protective paternalist	5.39 (*1.42*)	.165**	.66 (*.534*)	[–.39; 1.71]

Complementary class differentiation	4.66 (*1.34*)	–.015	–.50 (*.535*)	[–1.55; .55]

Subjective social class	5.34 (*2.86*)	.130**	.61 (*.534*)	[–.43; 1.66]

Objective social class	5.91 (*1.42*)	.054	.22 (*.262*)	[–.29; .74]


*Note*: ** *p ≤* .001; * *p ≤* .05.

In short, in this study, we aimed to explore which hierarchy-enhancing ideologies are strongly related to the perceived humanity gap between low- and high-SES groups. The results indicate that the tendency to perceive that some groups should have higher standings because of their superior natures (group dominance orientation), the belief that the world is a just place where individuals get what they deserve (SJ beliefs), and the explicit derogation of low-SES people as exploiting the welfare system and taking advantage of others (hostile classism) are ideological variables that are highly predictive of the differences in humanity between socioeconomic groups. In the following study, we aimed to expand these findings by (1) focusing on the role of each variable in the humanity gap and (2) incorporating attributional variables as mediating variables and policy preferences as distal outcomes of these ideological variables.

## Study 1

In this study, we aimed to expand previous findings by analysing the extent to which participants’ adherence to group dominance orientation, SJ beliefs, and hostile classism predict (1) the humanity gap between low- and high-SES groups, (2) attributions about poverty and wealth—namely, the tendency to blame low-SES groups for their plight and praise high-SES groups for their advantaged position—and (3) support for social change policies (i.e., redistributions preferences, progressive taxation, and welfare policies).^1^ In order to better understand this process, we take into account previous research (e.g., Sainz, Martínez, Rodríguez-Bailón & Moya, 2019; Sainz, Martínez et al., 2020) that identified that low- and high-SES perceived humanity are used to evaluate the causes of poverty and wealth (i.e., attributions about poverty and wealth), which lastly lead people to support or reject redistributions policies, progressive taxation systems, or social welfare policies. Thus, in the present study, we aimed to test a sequential mediational model in which the ideological variables act as antecedents of these redistribution initiatives via the perceived humanity gap and the attributional processes.^3^ The preregistration of the hypothesis can be found online: https://osf.io/e7gc5

## Method

### Participants and Procedure

We recruited participants using Prolific Academic services.^4^ The participants were paid 1£ for answering a four-minute study. The a priori sample size was originally computed for small–medium effect size (multiple regression, 10 predictors, 80% power, α = .05, *f*^2^ = .04) using G*Power analysis (Faul et al., 2009).^1^ A minimum of 416 participants was required. We attracted 523 participants, two of whom were excluded for failing to meet the preregistration inclusion criteria (e.g., by not being native Spanish speakers). The final sample comprised 521 Mexican participants (237 women, 282 men, and 2 others; *M*_age_ = 26.24, *SD* = 7.58).^4^ Once participants agreed to participate, they answered the following scales:

#### Ideological Orientation

According to the results of the Pilot Study, we included the items of the dominance orientation subfactor (α = .68), SJ beliefs (α = .85) and hostile classism (α =. 94) scales. Participants answered using a scale from 1 (*Completely disagree*) to 7 (*Completely agree*).

#### Humanity Gap

We computed the humanity gap between low-SES/poor groups (*r* = .901, *p* < .001) and high-SES/rich groups (*r* = .879, *p* < .001), as in the previous study.

#### Attributions About Poverty and Wealth

Participants answered the versions adapted for Spanish speakers (Sainz et al., 2022) of attributions about poverty (Cozzarelli et al., 2001) and wealth scales (Bullock et al., 2003), using a scale from 1 (*Completely disagree*) to 7 (*Completely agree*). We computed a blaming-low-SES-groups index by subtracting internal attributions about poverty (6 items, e.g., ‘Lack of thrift and proper money management,’ α = .88) from external attributions about poverty (5 items, e.g., ‘Prejudice and discrimination in hiring,’ α = .79) (see Sainz, Martínez, Rodríguez-Bailón & Moya, 2019; Sainz, Martínez et al., 2020). In the same way, we computed the index of praising high-SES groups by deducting the internal attributions about wealth items (8 items, e.g., ‘Ambition and personal drive,’ α = .90) from the external ones (5 items, e.g., ‘Better opportunities that result from being born into a well-off family,’ α = .76).

#### Support for Social Change Policies

To measure participants’ adherence to social change, we included three different scales (adapted from García-Castro et al., 2021; Sainz, Martínez, Rodríguez-Bailón & Moya, 2019; Sainz, Martínez et al., 2020). First, we measured participants’ levels of support for income redistribution policies (three items, e.g., ‘Income differences between those with more resources and those with fewer resources should be reduced’; α = .78). Second, we assessed participants’ level of support for a progressive taxation system (three items, e.g., ‘The government should impose higher taxes on people with more income’; α = .88). Third, we measured participants’ level of support for welfare policies (three items, e.g., ‘Poor people should receive more aid to improve their situations (unemployment, housing, etc.)’; α = .73). Participants answered using a scale from 1 (*Completely disagree*) to 7 (*Completely agree*).

Finally, as in the previous study, participants reported their subjective SES, objective SES, and demographic information.

## Results and Discussion

We first computed descriptive analysis, correlations between the variables (Table 2), differences in humanity between low-SES (*M* = 59.19; *SD* = 22.09) and high-SES groups (*M* = 66.01; *SD* = 21.01; *t*(520) = 7.83; *p* < .001; *d* = .32), and multiple regression analysis using the ideological variables as predictors of the dependent variables (Table 3). In general, the results highlight that the ideological variables positively predict the humanity gap, the tendency to blame low-SES groups and praise high-SES groups, and rejection of social change policies. Importantly, these results were unrelated to participants’ subjective and objective SES. There was only one exception, namely hostile classism, that did not predict the tendency to praise high-SES groups or the support for redistribution policies.

**Table 2:** Descriptive Statistics and Correlations Between Measures (Study 1).


	MEANS (*SDs*)	1	2	3	4	5	6	7	8	9	10	11

**1.** Group dominance	2.70 (*1.23*)	–	.455**	.477**	.326**	.463**	.421**	–.496**	–.311**	–.334**	.092*	.048

**2.** System justification	3.88 (*1.28*)		–	.558**	.319**	.679**	.670**	–.501**	–.380**	–.373**	.176**	–.043

**3.** Hostile classism	2.67 (*1.31*)			–	.383**	.570**	.406**	–.384**	–.331**	–.337**	.107*	–.058

**4.** Humanity gap	6.82 (*19.86*)				–	.320**	.345**	–.250**	–.259**	–.092*	.110*	.082^†^

**5.** Blaming low-SES groups	–1.35 (*1.76*)					–	.661**	–.508**	–.439**	–.434**	.132**	–.004

**6.** Praising high-SES groups	–1.08 (*1.87*)						–	–.559**	–.494**	–.352**	.169**	.004

**7.** Support for redistribution policies	4.88 (*1.51*)							–	.607**	.401**	–.103*	–.008

**8.** Support for progressive taxation	5.03 (*1.69*)								–	.297**	–.114*	–.013

**9.** Support for welfare policies	4.91 (*1.31*)									–	–.016	.036

**10.** Subjective social class	5.86 (*1.43*)										–	.399**

**11.** Objective social class	5.37 (*2.96*)											–


*Note*: SES = Socioeconomic status; ** *p ≤* .001; * *p ≤* .05; ^†^ *p ≤* .07.

**Table 3:** Multiple Regression Analysis of Ideological Variables and Dependent Variables (Study 1).


	HUMANITY GAP		BLAMING LOW-SES GROUPS		PRAISING HIGH-SES GROUPS		REDISTRIBUTION POLICIES		PROGRESSIVE TAXATION		WELFARE POLICIES	

	*F*_(5, 502)_ = 24.68**, *R^2^* = .189		*F*_(5, 500)_ = 112.36**, *R^2^* = .524		*F*_(5, 497)_ = 86.93**, *R^2^* = .461		*F*_(5, 494)_ = 51.53**, *R^2^* = .336		*F*_(5, 494)_ = 21.44**, *R^2^* = .170		*F*_(5, 494)_ = 22.60**, *R^2^* = .178	

	*β(SE)*	95% CI	*β(SE)*	95% CI	*β(SE)*	95% CI	*β(SE)*	95% CI	*β(SE)*	95% CI	*β* (SE)	95% CI

Group dominance	.158 (*.766*)**	[1.05; 4.06]	.127 (*.051*)**	[.08; .28]	.144 (*.059*)**	[.10; .33]	–.329 (*.053*)**	[–.50; –.30]	–.135 (*.066*)**	[–.31; –.06]	–.175 (*.051*)**	[–.29; –.09]

System justification	.114 (*.787*)*	[.22; 3.31]	.485 (*.053*)**	[.56; .76]	.595 (*.061*)**	[.75; .99]	–.318 (*.054*)**	[–.48; –.27]	–.229 (*.068*)**	[–.43; –.17]	–.220 (*.052*)**	[–.33; –.12]

Hostile classism	.251 (.*767*)**	[2.28; 5.29]	.247 (*.052*)**	[.23; .43]	.000 (*.059*)	[–.12; .12]	–.053 (*.053*)	[–.17; .04]	–.140 (*.066*)**	[–.31; –.05]	–.138 (*.051*)**	[–.24; –.04]

Subjective social class	.020 (*.620*)	[–.94; 1.50]	–.012 (*.042*)	[–.10; .07]	.049 (*.048*)	[–.03; .16]	–.003 (*.043*)	[–.09; .08]	–.035 (*.053*)	[–.15; .06]	.060 (*.041*)	[–.03; .14]

Objective social class	.085 (*.297*)^†^	[–.01; 1.16]	.026 (*.020*)	[–.02; .06]	.003 (*.023*)	[–.04; .05]	–.004 (*.021*)	[–.04; .04]	–.009 (*.026*)	[–.06; .05]	.005 (*.020*)	[–.04; .04]


*Note*: SES = Socioeconomic status; ** *p* ≤ .001; ^†^ *p* ≤ .09.

**Figure 1:**
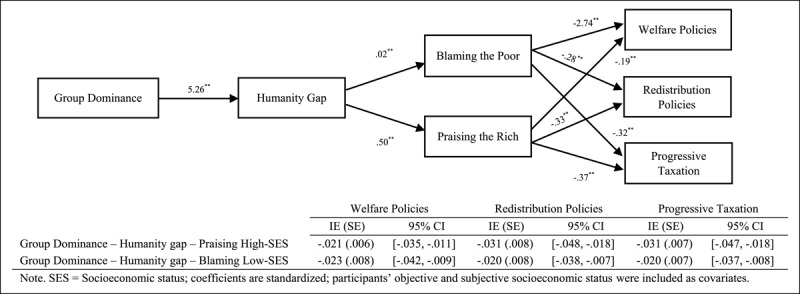
Several Sequential Mediational Analyses of Humanity Gap (Mediator 1) and Blaming Low-SES Groups (Mediator 2)/Praising High-SES Groups (Mediator 2) on Relationship between Group Dominance and Support for Social Change Policies (Study 1). Sequential Indirect Effects are Included in the Tables.

**Figure 2:**
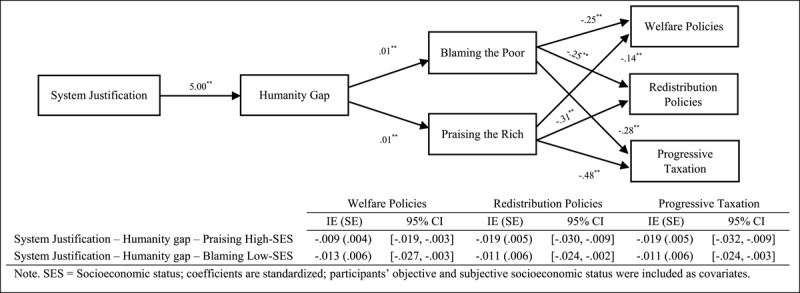
Several Sequential Mediational Analyses of Humanity Gap (Mediator 1) and Blaming Low-SES Groups (Mediator 2)/Praising High-SES Groups (Mediator 2) on Relationship between System Justification and Support for Social Change Policies (Study 1). Sequential Indirect Effects are Included in the Tables.

**Figure 3:**
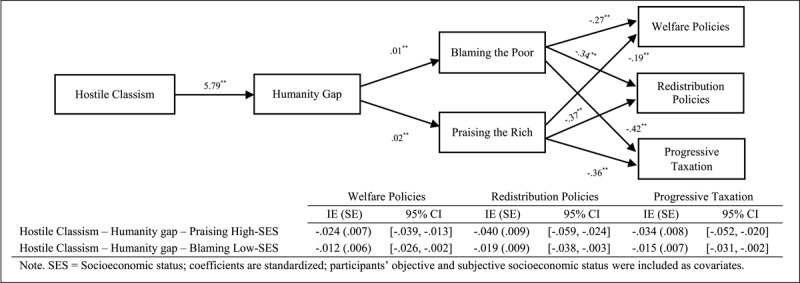
Several Sequential Mediational Analyses of Humanity Gap (Mediator 1) and Blaming Low-SES Groups (Mediator 2)/Praising High-SES Groups (Mediator 2) on Relationship between Hostile Classism and Support for Social Change Policies (Study 1). Sequential Indirect Effects are Included in the Tables.

Finally, we focused on testing sequential mediational analysis (Model 6, PROCESS, Hayes, 2013, 10,000 interactions, 95% confidence interval [CI]) by analysing the roles of the humanity gap (Mediator 1) and attributions of poverty and wealth (Mediator 2) in the relationship between the ideological variables and support for social change policies (Figures 1, 2, 3). Overall, the results indicate that the sequential mediational analyses of the humanity gap and the attributional processes are statistically significant. Specifically, the results show that the humanity gap and the attributional processes (i.e., blaming low-SES groups and praising high-SES groups) partially mediate the relationships between the ideological variables (namely group dominance, system justification and hostile classism), and the measures of social change (namely redistribution policies, progressive taxation system and welfare policies). With the two exceptions of (a) the lack of a direct effect (after including the mediators) of hostile classism on progressive taxation in the blaming-low-SES-groups (i.e., attributions about poverty) analysis and (b) the lack of a direct effect of system justification on progressive taxation in the praising-high-SES-groups (i.e., attributions about wealth) analysis, the results indicated two fully sequential mediational analyses (see supplementary materials for a full disclosure of the analyses).

In short, through this study, we provide evidence about the roles of group dominance orientation, SJ, and hostile classism in predicting the humanity gap, which, in turn, triggers attributional processes that, finally, lead to the rejection of social change policies. However, to acquire this evidence, we relied on correlational data; therefore, as a final step to test the causal influence of the ideological variables, we decided to implement an experimental procedure to manipulate these variables.

## Study 2 (A–C)

To confirm the ideological variables’ causal effect on individuals’ perceptions, we performed three experimental studies in which we indirectly manipulated social dominance (Study 2A), SJ beliefs (Study 2B), and hostile classism (Study 2C). On this issue, we should acknowledge the limitations that previous research highlights when manipulating participants’ ideological points of view in experimental settings. Previously used manipulations of these variables mainly rely on priming procedures that try to make certain mindsets (e.g., conservative ways of thinking). However, these manipulations sometimes exert a limited influence on participants’ ideological positionings during the experimental session (Kay et al., 2005; Madeira et al., 2019). Thus, we decided to test an alternative approach by using an experimental paradigm that, by asking participants to assume certain ideological positioning in a fictitious scenario, allows us to indirectly test how a high endorsement (vs. a low endorsement) of these ideological variables might influence the variables we are measuring. We assume that manipulating individuals’ ideology is a complex task practically unachievable in an experimental context. However, by following this paradigm we aim to show that there is a causal effect of different types of thinking regarding inter-group hierarchies on attributing different levels of humanity to advantages and disadvantage groups, attributions about poverty and wealth, and support for social change. Thus, we expected that a high endorsement (vs. a low endorsement) of the ideological variables would trigger: (1) a high perceived humanity gap, (2) a high tendency to blame low-SES groups and praise high-SES groups, and (3) low support for social change policies. Furthermore, as in Study 1, we aimed to confirm the sequential mediational analysis.^3^ The hypothesis’s preregistration can be found online: https://osf.io/9pzhy.

## Method

### Participants

We recruited a total of 631 Mexican participants using Prolific Academic services (1£/5-minute study).^4^ The a priori sample size was computed for small–medium effect size (ANOVA, 80% Power, α = .05, *f* ^2^ = .02) using G*Power analysis (Faul et al., 2009). A minimum of 200 participants per study was required. Final samples on each study were the following: 206 in Study 2A (103 women, 100 men, and 3 others; *M*_age_ = 25.03; *SD* = 5.74), 212 in Study 2B (115 women, 93 men, and 4 others; *M*_age_ = 24.06; *SD* = 5.63) and 213 in Study 2C (117 women, 95 men, and 1 other; *M*_age_ = 24.72; *SD* = 5.31). Once participants agreed to participate, they were presented with the following information:

### Ideological Orientation Manipulation

We told participants to imagine that they would be starting a new life in a society called Bimbola (Jetten et al., 2015; Sainz et al., 2021). Within this society, they would have a specific mindset regarding perceptions of others and understanding of social relationships. After being introduced to Bimbola, participants were randomly assigned to one of two possible conditions in each study (e.g., social dominance vs. non–social dominance in Study 2a). Then, we asked them to read a text including a brief description of how they were supposed to think within this society.^4^ We created these descriptions based on the original scales’ items (dominance orientation subfactor, SJ beliefs, and hostile classism subfactor) to simulate each ideological way of thinking. For example, participants in Study 2A’s social dominance condition read a text highlighting a dominance mind-set: ‘People like me believe that, in the society in which we live, some groups of people are simply inferior to other groups of people. For this reason, groups that are below others in society do not deserve to receive the same resources, benefits or rights as groups of people who are above or at the top of the social hierarchy.’

After the participants read the descriptions, we asked them to summarise their mindset in Bimbola in their own words to reinforce the manipulation and to answer two items (in each study) selected from the dominance orientation (Study 2A: *r* = .896, *p* < .001), SJ beliefs (Study 2B: *r* = .812, *p* < .001) and hostile classism (Study 2C: *r* = .791, *p* < .001) subscales, which acted as manipulation checks. Participants were asked to answer these items based on the way of thinking they were assigned in Bimbola (e.g., ‘Based on the text you read, to what extent do you think that some groups of people are simply inferior to other groups of people’ in Study 2A). Answers to these items ranged from 1 (*not at all*) to 7 (*completely*).

Once we presented participants with the experimental manipulation information, we asked them to answer the same scales as in previous studies based on the experimental condition.

#### Humanity Gap

We asked participants to rate the humanity of low-SES/poor groups (*r* = .904, *p* < .001) and that of high-SES/rich (*r* = .853, *p* < .001) groups—importantly—based on their experimental condition’s way of thinking (‘Taking into account your way of thinking in Bimbola, in general, how do you think the average members of the following groups are?’). We computed the same humanity gap index as in previous studies.

#### Attributions about Poverty and Wealth

Participants answered the blaming-low-SES-groups index (internal attributions [α = .92)) minus external attributions about poverty [α = .79]) and the praising-high-SES-groups index (internal attributions [α = .94] minus external attributions about wealth [α = .87]) based on their experimental condition’s way of thinking.

#### Support for Social Change Policies

Participants provided their levels of support for income redistribution policies (*r* = .864; *p* < .001),^5^ progressive taxation system (α = .85), and welfare policies (α = .89)—importantly—based on their experimental condition’s way of thinking.

Additionally, at the end of the study, we asked participants to provide their own personal views in real life by answering the dominance orientation (α = .76),^6^ SJ beliefs (α = .87), and hostile classism (α =. 96) scales. As in the previous studies, we also asked them to report their subjective SES, objective SES, and demographic information. Finally, we debriefed them and thanked them for their participation.

## Results and Discussion

We first confirmed that the manipulations worked properly given that participants correctly identified their assigned mind-sets. Participants showed differences in adherence to the inequality ideologies, according to the experimental condition (Study 2A: *M* = 5.85, *SD* = 1.88; Study 2B: *M* = 6.05, *SD* = 1.47; Study 2C: *M* = 5.85, *SD* = 1.73), and nonadherence to these ideologies in Bimbola (Study 2A: *M* = 1.65, *SD* = 1.20, *t* (204) = 19.09, *p* < .001, *d* = 2.66; Study 2B: *M* = 2.52, *SD* = 1.43, *t* (210) = 17.75 *p* < .001, *d* = 2.43; Study 2C: *M* = 1.83, *SD* = 1.79, *t* (210) = 13.21, *p* < .001, *d* = 2.28) when answering the manipulation checks.

Secondly, we performed analysis of covariance in each study to test the differences between the experimental conditions’ (adherence vs. nonadherence to the ideological variables; between-subjects comparison) effects on the included variables (i.e., humanity gap, blaming low-SES groups, praising high-SES groups, and support for social change policies). When performing these analyses, we controlled for participants’ real socioeconomic standings and their own ideological points of view (i.e., dominance orientation, SJ beliefs, and hostile classism). We did this to test our experimental manipulations’ influence without the effect of participants’ real positioning (Tables 4, 5, 6). Generally, each study’s results indicated that the experimental manipulations affected the dependent variables beyond the covariates. This implies that adherence to social dominance (vs. nonsocial dominance), SJ beliefs (vs. non-SJ beliefs), and hostile classism (vs. non–hostile classism) makes individuals more likely to perceive a large humanity gap, blame low-SES groups, praise high-SES groups, and object to social change policies. A few exceptions arise, such as in the case of some covariates that exert a significant, albeit minor, effect on the studies’ variables (e.g., individuals’ real hostile classism’s effects on the humanity gap). Thus, this analysis confirmed our hypothesised influence of ideological manipulation on the variables.

**Table 4:** Effects of Dominance Orientation Manipulations on Variables, Controlled for Participants’ Socioeconomic and Real Ideological Standings (Study 2A).


	HUMANITY GAP	BLAMING LOW-SES GROUPS	PRAISING HIGH-SES GROUPS	WELFARE POLICIES	REDISTRIBUTION POLICIES	PROGRESSIVE TAXATION

Main effect	*F*_(1, 202)_ = 351.28, *p* < .001, \[ \eta _p^2 = .624 \]	*F*_(1, 202)_ = 292.13, *p* < .001, \[ \eta _p^2 = .598 \]	*F*_(1, 202)_ = 59.02, *p <* .001, \[ \eta _p^2 = .231 \]	*F*_(1, 202)_ = 200.61, *p* < .001, \[ \eta _p^2 = .506 \]	*F*_(1, 202)_ = 303.10, *p* < .001, \[ \eta _p^2 = .607 \]	*F*_(1, 202)_ = 158.55, *p* < .001, \[ \eta _p^2 = .447 \]

	High-dominance: *M* = 73.59; *SD* = 26.70 Low-dominance: *M* = 6.88; *SD* = 23.39	High-dominance: *M* = 2.64; *SD* = 2.45 Low-dominance: *M* = 2.76; *SD* = 2.10	High-dominance: *M* = .64; *SD* = 2.03 Low-dominance: *M* = -1.68; *SD* = 2.20	High-dominance: *M* = 2.56; *SD* = 1.77 Low-dominance: *M* = 5.76; *SD* = 1.37	High-dominance: *M* = 2.06; *SD* = 1.60 Low-dominance: *M* = 5.85; *SD* = 1.44	High-dominance: *M* = 2.52; *SD* = 1.73 Low-dominance: *M* = 5.44; *SD* = 1.50

Covariates						

Subjective social class	*F*_(1, 202)_ = .135, *p* = .714, \[ \eta _p^2 = .001 \]	*F*_(1, 202)_ = 4.24, *p* = .041, \[ \eta _p^2 = .021 \]	*F*_(1, 202)_ = .46, *p* = .499, \[ \eta _p^2 = .002 \]	*F*_(1, 202)_ = 2.93, *p* = .089, \[ \eta _p^2 = .015 \]	*F*_(1, 202)_ = .02, *p* = .890, \[ \eta _p^2 = .000 \]	*F*_(1, 202)_ = .27, *p* = .605, \[ \eta _p^2 = .001 \]

Objective social class	*F*_(1, 202)_ = .00, *p* = .993, \[ \eta _p^2 = .000 \]	*F*_(1, 202)_ = 1.10, *p* = .306, \[ \eta _p^2 = .005 \]	*F*_(1, 202)_ = .55, *p* = .459, \[ \eta _p^2 = .003 \]	*F*_(1, 202)_ = .21, *p* = .649, \[ \eta _p^2 = .001 \]	*F*_(1, 202)_ = .40, *p* = .527, \[ \eta _p^2 = .002 \]	*F*_(1, 202)_ = 1.28, *p* = .260, \[ \eta _p^2 = .006 \]

Dominance orientation	*F*_(1, 202)_ = 1.35, *p* = .247, \[ \eta _p^2 = .007 \]	*F*_(1, 202)_ = 3.28, *p* = .071, \[ \eta _p^2 = .016 \]	*F*_(1, 202)_ = .19, *p* = .663, \[ \eta _p^2 = .001 \]	*F*_(1, 202)_ = .17, *p* = .681, \[ \eta _p^2 = .001 \]	*F*_(1, 202)_ = .02, *p* = .893, \[ \eta _p^2 = .000 \]	*F*_(1, 202)_ = .01, *p* = .937, \[ \eta _p^2 = .000 \]

System Justification	*F*_(1, 202)_ = .063, *p* = .802, \[ \eta _p^2 = .000 \]	*F*_(1, 202)_ = .98, *p* = .324, \[ \eta _p^2 = .005 \]	*F*_(1, 202)_ = .86, *p* = .355, \[ \eta _p^2 = .004 \]	*F*_(1, 202)_ = .822, *p* = .366, \[ \eta _p^2 = .004 \]	*F*_(1, 202)_ = .47, *p* = .492, \[ \eta _p^2 = .002 \]	*F*_(1, 202)_ = .24, *p* = .628, \[ \eta _p^2 = .001 \]

Hostile classism	*F*_(1, 202)_ = 6.64, *p* = .011, \[ \eta _p^2 = .033 \]	*F*_(1, 202)_ = 5.05, *p* = .026, \[ \eta _p^2 = .025 \]	*F*_(1, 202)_ = .07, *p* = .797, \[ \eta _p^2 = .000 \]	*F*_(1, 202)_ = .75, *p* = .388, \[ \eta _p^2 = .004 \]	*F*_(1, 202)_ = 1.30, *p* = .255, \[ \eta _p^2 = .007 \]	*F*_(1, 202)_ = .23, *p* = .634, \[ \eta _p^2 = .001 \]


**Table 5:** Effects of System Justification Manipulations on Variables, Controlled for Participants’ Socioeconomic and Real Ideological Standings (Study 2B).


	HUMANITY GAP	BLAMING LOW-SES GROUPS	PRAISING HIGH-SES GROUPS	WELFARE POLICIES	REDISTRIBUTION POLICIES	PROGRESSIVE TAXATION

Main effect	*F*_(1, 200)_ = 49.40, *P* < .001, \[ \eta _p^2 = .203 \]	*F*_(1, 200_ = 318.87, *p* < .001, \[ \eta _p^2 = .622 \]	*F*_(1, 200)_ = 320.27, *p* < .001, \[ \eta _p^2 = .623 \]	*F*_(1, 200)_ = 212.11, *p* < .001, \[ \eta _p^2 = .024 \]	*F*_(1, 200)_ = 276.92, *p* < .001, \[ \eta _p^2 = .588 \]	*F*_(1, 200)_ = 201.47, *p* < .001, \[ \eta _p^2 = .509 \]

	High-system justification: *M* = 53.37; *SD* = 27.14 Low-system justification: *M* = 20.47; *SD* = 34.22	High-system justification: *M* = 2.79; *SD* = 2.43 Low-system justification: *M* = -2.78; *SD* = 1.80	High-system justification: *M* = 2.32; *SD* = 2.01 Low-system justification: *M* = -2.73; *SD* = 1.87	High-system justification: *M* = 2.69; *SD* = 1.58 Low-system justification: *M* = 5.68; *SD* = 1.10	High-system justification: *M* = 2.33; *SD* = 1.44 Low-system justification: *M* = 5.69; *SD* = 1.32	High-system justification: *M* = 2.83; *SD* = 1.56 Low-system justification: *M* = 5.75; *SD* = 1.19

Covariates						

Subjective social class	*F*_(1, 200)_ = .10, *p* = .754, \[ \eta _p^2 = .001 \]	*F*_(1, 200)_ = .012, *p* = .911, \[ \eta _p^2 = .000 \]	*F*_(1, 200)_ = .00, *p* = .961, \[ \eta _p^2 = .000 \]	*F*_(1, 200)_ = .04, *p* = .845, \[ \eta _p^2 = .000 \]	*F*_(1, 200)_ = .31, *p* = .580, \[ \eta _p^2 = .002 \]	*F*_(1, 200)_ = .506, *p* = .478, \[ \eta _p^2 = .003 \]

Objective social class	*F*_(1, 200)_ = .48, *p* = .490, \[ \eta _p^2 = .002 \]	*F*_(1, 200)_ = .189, *p* = .664, \[ \eta _p^2 = .001 \]	*F*_(1, 200)_ = .76, *p* = .386, \[ \eta _p^2 = .004 \]	*F*_(1, 200)_ = .017, *p* = .896, \[ \eta _p^2 = .000 \]	*F*_(1, 200)_ = .02, *p* = .878, \[ \eta _p^2 = .000 \]	*F*_(1, 200)_ = .019, *p* = .889, \[ \eta _p^2 = .000 \]

Dominance orientation	*F*_(1, 200)_ = .29, *p* = .593, \[ \eta _p^2 = .001 \]	*F*_(1, 200)_ = .030, *p* = .863, \[ \eta _p^2 = .000 \]	*F*_(1, 200)_ = .37, *p* = .544, \[ \eta _p^2 = .002 \]	*F*_(1, 200)_ = .44, *p* = .510, \[ \eta _p^2 = .002 \]	*F*_(1, 200)_ = .07, *p* = .791, \[ \eta _p^2 = .000 \]	*F*_(1, 200)_ = .366, *p* = .546, \[ \eta _p^2 = .002 \]

System Justification	*F*_(1, 200)_ = 3.77, *p* = .054, \[ \eta _p^2 = .019 \]	*F*_(1, 200)_ = 1.10, *p* = .297, \[ \eta _p^2 = .006 \]	*F*_(1, 200)_ = 1.31, *p* = .254, \[ \eta _p^2 = .007 \]	*F*_(1, 200)_ = 2.18, *p* = .141, \[ \eta _p^2 = .011 \]	*F*_(1, 200)_ = 1.37, *p* = .244, \[ \eta _p^2 = .007 \]	*F*_(1, 200)_ = 1.84, *p* = .177, \[ \eta _p^2 = .009 \]

Hostile classism	*F*_(1, 200)_ = 6.00, *p* = .015, \[ \eta _p^2 = .030 \]	*F*_(1, 200)_ = 7.40, *p* = .007, \[ \eta _p^2 = .037 \]	*F*_(1, 200)_ = 1.67, *p* = .197, \[ \eta _p^2 = .009 \]	*F*_(1, 200)_ = 1.66, *p* = .199, \[ \eta _p^2 = .008 \]	*F*_(1, 200)_ = 2.06, *p* = .153, \[ \eta _p^2 = .010 \]	*F*_(1, 200)_ = 2.90, *p* = .090, \[ \eta _p^2 = .090 \]


**Table 6:** Effects of Hostile Classism Manipulations on Variables, Controlled for Participants’ Socioeconomic and Real Ideological Standings (Study 2C).


	HUMANITY GAP	BLAMING LOW-SES GROUPS	PRAISING HIGH-SES GROUPS	WELFARE POLICIES	REDISTRIBUTION POLICIES	PROGRESSIVE TAXATION

Main effect	*F*_(1, 202)_ = 133.1, *p* < .001, \[ \eta _p^2 = .404 \]	*F*_(1, 202)_ = 149.22, *p* < .001, \[ \eta _p^2 = .432 \]	*F*_(1, 202)_ = 49.75, *p* < .001, \[ \eta _p^2 = .202 \]	*F*_(1, 202)_ = 95.40, *p* < .001, \[ \eta _p^2 = .{\mathrm{327}} \]	*F*_(1, 202)_ = 72.64, *p* < .001, \[ \eta _p^2 = .{\mathrm{27}}0 \]	*F*_(1, 202)_ = 105.57, *p* < .001, \[ \eta _p^2 = .{\mathrm{350}} \]

	Hostile classism: *M* = 66.04; *SD* = 31.91 Non-hostile classism_: *M* = 14.72; *SD* = 27.28	Hostile classism: *M* = 3.42; *SD* = 2.33 Non-hostile classism: *M* = 1.26; *SD* = 2.90	Hostile classism: *M* = 1.68; *SD* = 2.00 Non-hostile classism: *M* = .53; *SD* = 2.22	Hostile classism: *M* = 2.51; *SD* = 1.58 Non-hostile classism: *M* = 4.85; *SD* = 1.77	Hostile classism: *M* = 2.06; *SD* = 1.59 Non-hostile classism: *M* = 4.21; *SD* = 1.94	Hostile classism: *M* = 2.31; *SD* = 1.45 Non-hostile classism: *M* = 4.66; *SD* = 1.76

Covariates						

Subjective social class	*F*_(1, 202)_ = .06, *p* = .813, \[ \eta _p^2 = .000 \]	*F*_(1, 202)_ = 1.40, *p* = .237, \[ \eta _p^2 = .00{\mathrm{7}} \]	*F*_(1, 202)_ = .77, *p* = .380, \[ \eta _p^2 = .00{\mathrm{4}} \]	*F*_(1, 202)_ = .19, *p* = .664, \[ \eta _p^2 = .00{\mathrm{1}} \]	*F*_(1, 202)_ = .41, *p* = .524, \[ \eta _p^2 = .00{\mathrm{2}} \]	*F*_(1, 202)_ = .12, *p* = .731, \[ \eta _p^2 = .00{\mathrm{1}} \]

Objective social class	*F*_(1, 202)_ = .00, *p* = .961, \[ \eta _p^2 = .000 \]	*F*_(1, 202)_ = .54, *p* = .463, \[ \eta _p^2 = .00{\mathrm{3}} \]	*F*_(1, 202)_ = .01, *p* = .932, \[ \eta _p^2 = .000 \]	*F*_(1, 202)_ = 1.56, *p* = .213, \[ \eta _p^2 = .00{\mathrm{8}} \]	*F*_(1, 202)_ = .01, *p* = .941, \[ \eta _p^2 = .000 \]	*F*_(1, 202)_ = .104, *p* = .747, \[ \eta _p^2 = .00{\mathrm{1}} \]

Dominance orientation	*F*_(1, 202)_ = 4.16, *p* = .043, \[ \eta _p^2 = .0{\mathrm{21}} \]	*F*_(1, 202)_ = 4.54, *p* = .034, \[ \eta _p^2 = .0{\mathrm{23}} \]	*F*_(1, 202)_ = .92, *p* = .338, \[ \eta _p^2 = .00{\mathrm{5}} \]	*F*_(1, 202)_ = 5.72, *p* = .018, \[ \eta _p^2 = .028 \]	*F*_(1, 202)_ = 3.03, *p* = .083, \[ \eta _p^2 = .0{\mathrm{15}} \]	*F*_(1, 202)_ = 6.76, *p* = .010, \[ \eta _p^2 = .0{\mathrm{33}} \]

System Justification	*F*_(1, 202)_ = .00, *p* = .968, \[ \eta _p^2 = .000 \]	*F*_(1, 202)_ = .31, *p* = .580, \[ \eta _p^2 = .00{\mathrm{2}} \]	*F*_(1, 202)_ = .17, *p* = .679, \[ \eta _p^2 = .00{\mathrm{1}} \]	*F*_(1, 202)_ = .83, *p* = .363, \[ \eta _p^2 = .00{\mathrm{4}} \]	*F*_(1, 202)_ = .40, *p* = .528, \[ \eta _p^2 = .00{\mathrm{2}} \]	*F*_(1, 202)_ = .002, *p* = .968, \[ \eta _p^2 = .000 \]

Hostile classism	*F*_(1, 202)_ = .06, *p* = .809, \[ \eta _p^2 = .000 \]	*F*_(1, 202)_ = .18, *p* = .669, \[ \eta _p^2 = .00{\mathrm{1}} \]	*F*_(1, 202)_ = .45, *p* = .502, \[ \eta _p^2 = .00{\mathrm{2}} \]	*F*_(1, 202)_ = .38, *p* = .540, \[ \eta _p^2 = .00{\mathrm{2}} \]	*F*_(1, 202)_ = 1.92, *p* = .168, \[ \eta _p^2 = .01{\mathrm{0}} \]	*F*_(1, 202)_ = 1.29, *p* = .257, \[ \eta _p^2 = .007 \]


**Figure 4:**
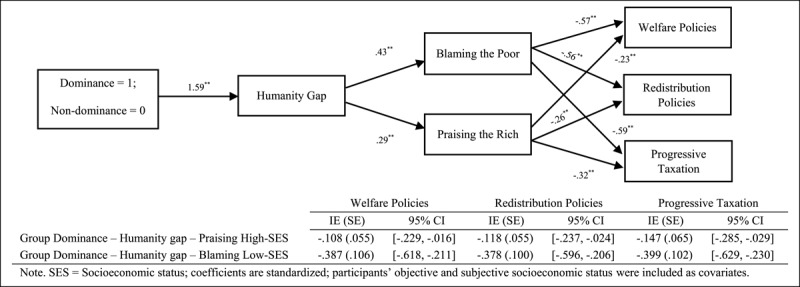
Several Sequential Mediational Analyses of Humanity Gap (Mediator 1) and Blaming Low-SES Groups (Mediator 2)/Praising High-SES Groups (Mediator 2) on Relationship between Group Dominance (Manipulated) and Support for Social Change Policies (Study 2A). Sequential Indirect Effects are Included in the Tables.

**Figure 5:**
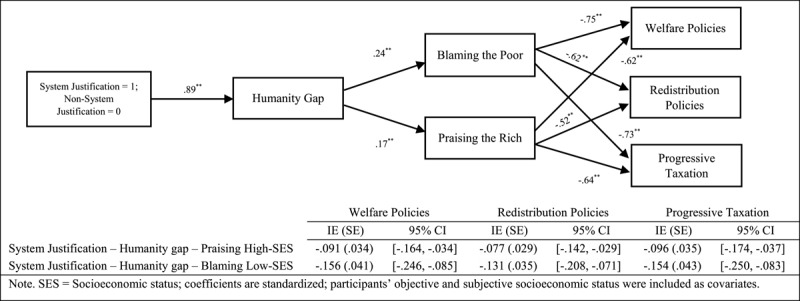
Several Sequential Mediational Analyses of Humanity Gap (Mediator 1) and Blaming Low-SES Groups (Mediator 2)/Praising High-SES Groups (Mediator 2) on Relationship between System Justification (Manipulated) and Support for Social Change Policies (Study 2B). Sequential Indirect Effects are Included in the Tables.

**Figure 6:**
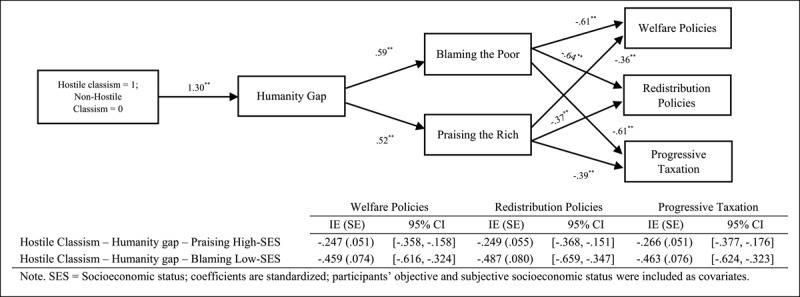
Several Sequential Mediational Analyses of Humanity Gap (Mediator 1) and Blaming Low-SES Groups (Mediator 2)/Praising High-SES Groups (Mediator 2) on Relationship between Hostile Classism (Manipulated) and Support for Social Change Policies (Study 2C). Sequential Indirect Effects are Included in the Tables.

Finally, following results of Study 1, we tested the sequential mediational analysis (Model 6; PROCESS; Hayes, 2013; 10,000 interactions; 95% CI) using the humanity gap (Mediator 1) and blaming low-SES groups and praising high-SES groups (Mediator 2) in the relationship between the ideological variables (1 = adherence to the variables, 0 = nonadherence to the variables) and support for social change policies (Figures 4, 5, 6).^4^ Overall, our results support the sequential mediating effect of the humanity gap and attributional processes (blaming low-SES groups or praising high-SES groups) on the relationship between the ideological variables and support for social change policies. This effect implies that the ideological variables seem to trigger the differences in the humanisation of the two groups, which lastly created the perception that low- and high-SES groups are responsible for their economic standing and, thus, undeserving or deserving of help via social policies or income redistribution. Furthermore, we should acknowledge that the direct effect remains significant in some of the mediational analyses (i.e., partial mediational results) showing the relevance of both the ideological manipulations and the mediators included in the model (see supplementary materials for a full disclosure of the analyses).

In short, despite the indirect nature of our manipulations, these studies’ results (1) seem to confirm the ideological variables’ causal effect on the perceived humanity gap between low- and high-SES groups, attributional processes, and support for social change policies and (2) confirm the mediational role of the humanity gap and of poverty and wealth attributions in ideology’s effect on support for social change policies.

## General Discussion

Our aim in the present project was to test the influence of certain hierarchy-enhancing ideological variables, such as social dominance, SJ beliefs, and hostile classism, on the perceived humanity gap between low- and high-SES groups that contributes to maintaining economic inequality. To do so, we carried out two correlational and three experimental studies addressing how these ideological variables act as antecedents of the humanity gap, attributions of poverty and wealth, and support for social change policies. Generally, our results indicate that these three ideological variables exert effects on the humanity assigned to advantage and disadvantage groups, how people understand socioeconomic differences, and on their support for social change aimed at reducing differences between groups.

First, evidence highlights the ideologies’ roles in the attribution of humanity. Specifically, our results indicate that the support for social hierarchies that legitimize the superiority of some groups above others, the perception that the economic systems is fair and hostile classist attitudes against low-SES groups are consistent predictors of the humanity gap between low- and high-SES groups. This is a valuable finding given that previous research has mainly analysed the role of (de)humanisation of low- and high-SES groups separately, without comparing them together in a joint analysis (Sainz, Loughnan et al., 2020; Sainz, Martínez et al., 2020; Sainz, Martínez, Rodríguez-Bailón & Moya, 2019). This seems to be a key point as jointly analysing humanisation of both low- and high-SES groups provides complementary evidence that could be useful to understand what triggers the gap and based on that, to design interventions aimed at reducing the perceived humanity gap between socioeconomic groups.

Second, the present project highlights certain ideological variables’ roles as antecedents of attributional processes of poverty and wealth and support for social change policies (Hunt & Bullock, 2016; Rodríguez-Bailón et al., 2017). In this regard, our results confirm that ideological perspectives impact on how people understand and interpret socioeconomic differences. Specifically, variables, such as classism or group dominance, influence people’s perceptions about poverty and low-SES groups (e.g., attribution about poverty, support for social policies), whereas others, such as system justification, are more closely related to the perception of high-SES groups or exert more influence on the rejection of policies aimed to directly increase taxes on wealthy groups. Additionally, our results confirm that (de)humanisation triggers the justification of socioeconomic differences. In this regard, (de)humanising low- and high-SES groups has been found to influence attributional processes about poverty and wealth that ultimately lead to justifying support or rejection of social change policies (Sainz, Loughnan et al., 2020; Sainz, Martínez et al., 2020; Sainz, Martínez, Rodríguez-Bailón & Moya, 2019).

Limitations apply to the present project. On this issue, we should acknowledge the limitations of manipulating participants’ stable and long-standing attitudes, such as their ideological positioning. Previous research mainly implemented priming procedures to make salient certain social norms and ways of thinking using sentence completion tasks (Madeira et al., 2019) or fake newspaper articles (e.g., Kay et al., 2005). These procedures, despite being a direct way of manipulating these variables, have limited and short influences on participants’ ways of thinking. For these reasons, we opted to implement a more indirect procedure using the Bimbola paradigm (Jetten et al., 2015), which has been successfully implemented on similar projects addressing socioeconomic variables (Sainz et al., 2021) and stereotyping of socioeconomic groups (Tanjitpiyanond et al., 2022). By using this paradigm, we have shown that different ways of thinking regarding groups hierarchies have a causal effect on dehumanization and support for social change. Despite the fact that we recognize the limitations of this procedure we highlight a) the similarities between the results in the correlational and the experimental studies as well as b) the fact that our pattern of results is in line with our hypothesis. This seems to reinforce the usefulness of this novel strategy to make salient different ideological points of view. Nevertheless, future studies could rely on longitudinal analysis measuring individuals’ attitudes to complement the results pattern we identified in our cross-sectional studies. Another limitation of this work is related to our samples. Participants lived in Mexico, an extremely unequal context where differences between low- and high-SES people are quite explicit. Future studies could test the generalisation of our results in other more egalitarian contexts, where differences between low- and high-SES groups are more subtle or less accepted.

Future studies could also deepen in the understanding of how individuals who adhere to hierarchy-enhancing variables (vs. more egalitarian individuals) ascribe humanity to low- and high-SES groups. On this issue, some previous research has considered that politically conservative individuals are uniquely prone to develop negative attitudes towards other groups; whereas other perspectives consider that there is some kind of symmetrical tendency in intergroup bias among conservative and nonconservative individuals (Crawford & Brandt, 2020; Jost, 2017). This latter perspective implies an equivalence in the tendency to hold bias against other groups with different values from those of one’s own group among both conservative (i.e., against disadvantaged or powerless groups) and nonconservative groups (i.e., against advantaged or powerful groups). This issue, as far as we acknowledge, has not been addressed in the dehumanisation literature. In this sense, it might be possible that individuals who adhere to hierarchy-enhancing variables have the tendency to dehumanise low-SES groups and humanising high-SES groups, whereas the opposite can be expected among individuals who do not adhere to hierarchy-enhancing variables. This hypothesis could be tested in future studies to increase the understanding of how ideological variables might shape the (de)humanisation of groups based on SES. In conclusion, hierarchy-enhancing ideological variables shape the humanization gap between low- and high-SES groups, which affect attributional processes of poverty and wealth and, in consequence, individuals’ support for social change.

## Data accessibility statement

Data and materials of this project can be found online at https://osf.io/c7qp3/.

## Additional File

The additional file for this article can be found as follows:

10.5334/irsp.753.s1 Supplemental File. Supplementary Online Materials.

## Footnotes

To simplify the project’s structure, we slightly deviate from the preregistration analysis by mainly focusing on the analysis corresponding with the humanity gap between low- and high-SES groups, without additionally presenting the analysis for low- and high-SES groups separately in Studies 1 to 3C. Furthermore, in Study 1, we simplify the results’ presentation by including uniquely the most suitable ideological predictors in the main analysis. However, full disclosure of the studies’ data and analysis can be found in the supplementary materials.

Data from the study are from a previous project that can be found at the following link: https://osf.io/c2et3/.

To simplify the structure of the project we deviated from the preregistration as we decided to compute indexes (i.e., Blazing the poor) when analysing the attributional processes related to poverty and wealth by following similar procedures that can be found in previous research (e.g., Sainz, Martínez et al., 2020).

See supplementary materials for a full description of the samples in the studies.

See supplementary materials for a full description of the manipulations and alternative analysis in Studies 2A–2C.

One item was excluded from this scale because it lowered the scale’s reliability.
