# Supplementary material for: Group Dominance, System Justification, and Hostile Classism: The Ideological Roots of the Perceived Socioeconomic Humanity Gap That Upholds the Income Gap
Source: Int Rev Soc Psychol. 2023 Jun 8;36:7. doi: 10.5334/irsp.753 (PMC12372804; doi:10.5334/irsp.753)
Supplement: Supplemental File. — Supplementary Online Materials. [file irsp-36-753-s1.pdf]

Group Dominance, System Justification, and Hostile Classism: The Ideological Roots of the Perceived Socioeconomic Humanity Gap That Upholds the Income Gap

Supplementary Online Materials

In the following pages you will find full disclosure of the analysis, tables, figures, and materials that we used in the present project. Part of the following information was pre-registered. However, with the aim to simplify the manuscript we included the additional information in the following document instead of in the main text. See the organization of the supplementary materials:

A) Full description of the participant's details in the studies.

B) Supplementary details and analysis of Pilot Study.

C) Supplementary details and analysis of Study 1.

D) Supplementary details and analysis of Studies 2a to 2c.

E) Supplementary details of the mediational analyses of Studies 1 to 2c

A) Full description of the participants details in the studies.

Table SM-1. *Description of the sample characteristics in the studies.*

	Pilot Study	Study 1	Study 2a	Study 2b	Study 2c
Participant's sex:	Woman = 314, Men = 445, Other = 6	Women = 237, Men = 282, Other = 2	Women = 103, Men = 100, Other = 3	Women = 115, Men = 93, Other = 4	Women = 117, Men = 95, Other = 1
Participants age	*Mean* = 26.01; *SD* = 7.37	*Mean* = 26.24; *SD* = 7.58	*Mean* = 25.03; *SD* = 5.74	*Mean* = 24.06; *SD* = 5.63	*Mean* = 24.72; *SD* = 5.31
Participant`s subjective socioeconomic status	*Mean* = 5.91; *SD* = 1.42	*Mean* = 5.86; *SD* = 1.43	*Mean* = 6.03; *SD* = 1.44	*Mean* = 6.04; *SD* = 1.25	*Mean* = 6.03; *SD* = 1.40
Participant`s objective socioeconomic status	*Mean* = 9,766.50; *SD* = 21,513.33	*Mean* = 12,565.79; *SD* = 56,468	*Mean* = 10,562.70; *SD* = 11,972.62	*Mean* = 11,233.48; *SD* = 27,209.00	*Mean* = 11,774.34; *SD* = 23,379.84

Note. Non grouped objective socioeconomic status variable (income/household members).

B) Supplementary details and analysis of Pilot Study.

In the following pages we included full disclosure of the analysis performed in the Pilot Study. Some of the following information was originally planned to be included in the main text. However, aiming to simplify the manuscript we deviate from our pre registration and include this information in the following pages as supplementary materials.

Table SM-2. *Full Descriptive Statistics and Correlations Between the Measures in the Pilot Study.*

	Mean (*SD*)	1	2	3	4	5	6	7	8	9	9.1	9.2	10	11
1. Group dominance	2.75 (*1.24*)	-	.602**	.402**	.293**	.461**	.522**	.047	-.128**	.382**	-.211**	.156**	.156**	.092*
2. Anti-egalitarianism	2.47 (*1.14*)		-	.357**	.274**	.562**	.451**	-.065	-.153**	.255**	-.116**	.128**	.102**	.035
3. System justification	3.94 (*1.26*)			-	.422**	.367**	.573**	.276**	.136**	.363**	-.129**	.218**	.183**	-.079*
4. Political orientation	3.73 (*1.14*)				-	.254**	.301**	.149**	.015	.240**	-.068	.161**	.099**	.022
5. Tolerance to inequality	2.12 (*0.97*)					-	.413**	-.130**	-.120**	.212**	-.128**	.076*	.070	-.035
6. Hostile classism	2.74 (*1.34*)						-	.254**	.043	.438**	-.284**	.137**	.084*	-.025
7. Protective paternalist	5.39 (*1.42*)							-	.311**	.165**	-.031	.127**	.051	-.018
8. Complementary class differentiation	4.66 (*1.34*)								-	-.015	.031	.017	-.015	-.119**
9. Humanity gap	6.33 (*20.79*)									-	-.470**	.489**	.130**	.054
9.1. Low-SES humanity	59.53 (*21.55*)										-	.540**	.020	.009
9.2. High-SES humanity	65.86 (*21.81*)											-	.143**	.060
10. Subjective social class	5.91 (*1.42*)												-	.425**
11. Objective social class	5.34 (*2.86*)													-

Note. SES = Socioeconomic status; ** $p \leq .001$; * $p \leq .05$.

Table SM-3. *Regression Analysis of each Ideological Variables predicting, individually, the Attribution of Humanity to Low-, High-SES groups and the Humanity Gap in the Pilot Study.*

	Low-SES humanity		High-SES humanity		Humanity gap	
	β (SE)	95% CI	*β (SE)*	95% CI	*β (SE)*	95% CI
Group dominance	F (3, 746) = 12.69**, R^2 = .045		F (3, 746) = 10.11**, R^2 = .035		F (3, 746) = 45.41**, R^2 = .151	
	-3.88 (*.631*)**	[-5.12; -2.64]	.135 (*.642*)**	[1.12; 3.64]	.373 (*.573*)**	[5.14; 7.39]
Anti-egalitarianism	F (3, 746) = 3.49*, R^2 = .010		F (3, 746) = 9.05**, R^2 = .031		F (3, 746) = 21.20**, R^2 = .075	
	-.117 (*.694*)**	[-.80; 1.62]	.118 (*.695*)**	[.90; 3.63]	.246 (*.645*)**	[3.22; 5.76]
System justification	F (3, 746) = 4.54*, R^2 = .014		F (3, 746) = 17.43**, R^2 = .062		F (3, 746) = 43.12**, R^2 = .144	
	-.137 (*.643*)**	[-3.62; -1.09]	.217 (*.635*)**	[2.52; 5.02]	.371 (*.576*)**	[4.99; 7.26]
Political orientation	F (3, 746) = 1.02, R^2 = .004		F (3, 746) = 11.88**, R^2 = .042		F (3, 746) = 18.93**, R^2 = .067	
	.022 (*.621*)†	[-.88; 1.56]	.157 (*.688*)**	[1.64; 4.34]	.229 (*.645*)**	[2.90; 5.43]
Tolerance to inequality	F (3, 746) = 4.17*, R^2 = .013		F (3, 746) = 6.68**, R^2 = .022		F (3, 746) = 16.12**, R^2 = .057	
	-.128 (*.805*)**	[-4.40; -1.24]	.070 (.811)†	[-.04; 3.15]	.206 (*.757*)**	[2.89; 5.86]
Hostile classism	F (3, 746) = 23.46**, R^2 = .083		F (3, 746) = 9.61**, R^2 = .033		F (3, 746) = 66.03**, R^2 = .207	
	-.295 (*.566*)**	[-5.86; -3.63]	.127 (*.589*)**	[.91; 3.22]	.440 (*.507*)**	[5.81; 7.80]
Protective paternalist	F (3, 746) = .322, R^2 = .001		F (3, 746) = 8.94**, R^2 = .031		F (3, 746) = 11.10**, R^2 = .039	
	-.032 (*.558*)	[-1.58; .61]	.116 (*.555*)**	[.69; 2.87]	.155 (*.526*)**	[1.24; 3.30]
Complementary class differentiation	F (3, 746) = .832, R^2 = .001		F (3, 746) = 5.58**, R^2 = .018		F (3, 746) = 4.67*, R^2 = .015	
	.030 (*.595*)	[-.68; 1.66]	.025 (*.597*)	[-.77; 1.57]	-.005 (*.568*)	[-1.20; 1.03]

Note. Beta coefficients are standardized. Analysis controlling by participants subjective and objective socioeconomic status (SES); ** $p \leq .001$; * $p \leq .05$; † $p \leq .091$.

Table SM-4. *Multiple Regression Analysis of Ideological Variables on the Attribution of Humanity to Low- and High-SES groups, controlled by participants' socioeconomic status, in the Pilot Study.*

	Low-SES humanity $F_{(10, 739)} = 8.36^{**}$, $R^2 = .089$		High-SES humanity $F_{(10, 739)} = 6.34^{**}$, $R^2 = .067$		Humanity gap $F_{(10, 739)} = 25.32^{**}$, $R^2 = .245$	
	β (*SE*)	95% CI	β (*SE*)	95% CI	β (*SE*)	95% CI
Group dominance	-.140 (*.839*)**	[-4.09; -.80]	.051 (*.860*)	[-.79; 2.59]	.199 (*.735*)**	[1.90; 4.79]
Anti-egalitarianism	.091 (*.929*)†	[-.10; 3.55]	.051 (*.953*)	[-.89; 2.85]	-.041 (*.814*)	[-2.34; .86]
System justification	.048 (*.826*)	[-.81; 2.44]	.163 (*.847*)**	[1.17; 4.50]	.123 (*.724*)**	[.60; 3.44]
Political orientation	.025 (*.741*)	[-.99; 1.92]	.072 (*.760*)†	[-.12; 2.86]	.050 (*.649*)	[-.37; 2.18]
Tolerance to inequality	-.006 (*.995*)	[-2.08; 1.83]	-.031 (*1.021*)	[-2.71; 1.30]	-.027 (*.873*)	[-2.28; 1.14]
Hostile classism	-.304 (*.778*)**	[-6.41; -3.36]	-.041 (*.798*)	[-2.23; .90]	.273 (*.682*)**	[2.88; 5.56]
Protective paternalist	.028 (*.609*)	[-.77; 1.62]	.070 (*.624*)†	[-.14; 2.31]	.045 (*.534*)	[-.39; 1.71]
Complementary class differentiation	.025 (*.610*)	[-.79; 1.60]	-.006 (*.626*)	[-1.32; 1.13]	-.032 (*.535*)	[-1.55; .55]
Subjective social class	.046 (*.609*)	[-.49; 1.91]	.085 (*.625*)*	[.10; 2.55]	.042 (*.534*)	[-.43; 1.66]
Objective social class	-.002 (*.299*)	[-.60; .57]	.027 (*.306*)	[-.40; .81]	.031 (*.262*)	[-.29; .74]

Note. SES = Socioeconomic status; ** $p \leq .001$; * $p \leq .05$; † $p \leq .083$.

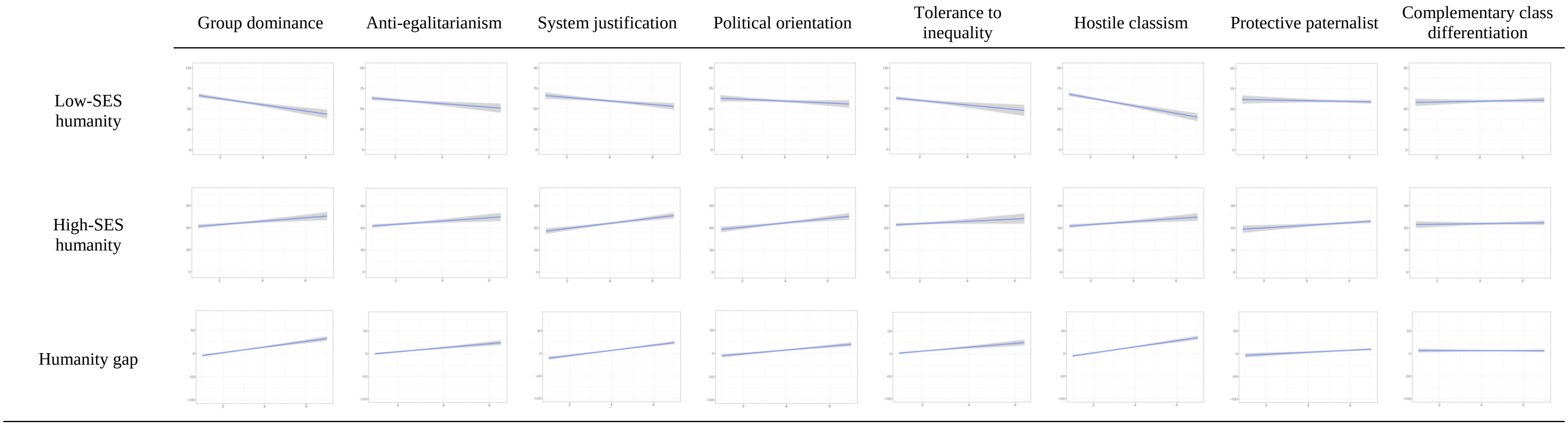

Figure SM-1. *Plots with Regression Coefficients of each Ideological Variable predicting the Attribution of Humanity to Low- and High-SES groups as well as the humanity gap, while controlling by participants subjective and objective socioeconomic status, in the Pilot Study.*

C) Supplementary details and analysis of Study 1.

In the following pages we included full disclosure of the analysis performed in Study 1. Some of the following information was expected to be included in the main text. However, aiming to simplify the manuscript we deviate from our pre registration and include this information in the following pages as supplementary materials.

Table SM-5. *Full Descriptive Statistics and Correlations Between the Measures included in the Study 1.*

	Mean (*SD*)	1	2	3	4	5	6	7	8	9	9.1	9.2	10	10.1	10.2	11	11.1	11.2	12	13	14	15	16
1. Group dominance	2.70 (*1.23*)	-	.574**	.455**	.260**	.468**	.477**	.126**	-.063	.326**	-.235**	.061	.463**	.384**	-.366**	.421**	.375**	-.214**	-.496**	-.311**	-.334**	.092*	.048
2. Anti-egalitarianism	2.49 (*1.15*)		-	.419**	.260**	.572**	.371**	.040	-.062	.176**	-.088*	.074†	.488**	.361**	-.438**	.378**	.277**	-.293**	-.503**	-.324**	-.403**	.043	-.046
3. System justification	3.88 (*1.28*)			-	.374**	.499**	.558**	.232**	.108*	.319**	-.142**	.153**	.679**	.631**	-.372**	.670**	.640**	-.303**	-.501**	-.380**	-.373**	.176**	-.043
4. Political orientation	3.77 (*1.11*)				-	.250**	.287**	.198**	.034	.197**	-.066	.116**	.341**	.328**	-.180**	.411**	.378**	-.169**	-.339**	-.395**	-.277**	.102*	-.019
5. Tolerance to inequality	2.15 (*1.02*)					-	.341**	-.009	-.073†	.203**	-.190**	-.008	.499**	.366**	-.458**	.428**	.313**	-.333**	-.501**	-.339**	-.394**	.08†	-.049
6. Hostile classism	2.67 (*1.31*)						-	.355**	.043	.383**	-.280**	.067	.570**	.607**	-.247**	.406**	.423**	-.111*	-.384**	-.331**	-.337**	.107*	-.058
7. Protective paternalist	5.38 (*1.58*)							-	.227**	.215**	-.064	.136**	.177**	.255**	.024	.257**	.338**	.045	-.129**	-.113*	-.051	.045	.011
8. Complementary class differentiation	4.54 (*1.27*)								-	-.070	.056	-.007	-.063	.044	.195**	.028	.130**	.142**	.058	.059	.097*	.046	-.073†
9. Humanity gap	6.82 (*19.86*)									-	-.503**	.417**	.320**	.309**	-.191**	.345**	.351**	-.088*	-.250**	-.259**	-.092*	.110*	.082†
9.1. Low-SES humanity	59.19 (*22.09*)										-	.576**	-.203**	-.191**	.150**	-.121**	-.124**	.004	.124**	.134**	.083†	.032	-.031
9.2. High-SES humanity	66.01 (*21.01*)											-	.089*	.091*	-.023	.200**	.202**	-.08†	-.105*	-.104*	.000	.138**	.044
10. Blaming low-SES	-1.35 (*1.76*)												-	.857**	-.637**	.661**	.564**	-.412**	-.508**	-.439**	-.434**	.132**	-.004
10.1. Internal attributions about poverty	3.69 (*1.48*)													-	-.187**	.543**	.593**	-.153**	-.383**	-.362**	-.325**	.130**	-.014
10.2. External attributions about poverty	5.55 (*1.09*)														-	-.467**	-.228**	.520**	.424**	.329**	.384**	-.082†	-.007
11. Praising high-SES	-1.08 (*1.87*)															-	.876**	-.610**	-.559**	-.494**	-.352**	.169**	.004
11.1. Internal attributions about wealth	4.54 (*1.34*)																-	-.201**	-.465**	-.403**	-.284**	.166**	.016
11.2. External attributions about wealth	5.82 (*0.93*)																	-	.319**	.266**	.236**	-.056	.023
12. Support for redistribution policies	4.88 (*1.51*)																		-	.607**	.401**	-.103*	-.008
13. Support for progressive taxation	5.03 (*1.69*)																			-	.297**	-.114*	-.013
14. Support for welfare policies	4.91 (*1.31*)																				-	-.016	.036
15. Subjective social class	5.86 (*1.43*)																					-	.399**
16. Objective social class	5.37 (*2.96*)																						-

Note. SES = Socioeconomic status; ** $p \le .001$; * $p \le .05$; † $p \le .099$.

Table SM-6. *Multiple Regression Analysis of Ideological Variables on the Attribution of Humanity to Low-, High-SES groups and the Humanity Gap in Study 1.*

	Low-SES humanity $F_{(10, 497)} = 7.40^{**}$, $R^2 = .112$		High-SES humanity $F_{(10, 497)} = 3.67^{**}$, $R^2 = .050$		Humanity gap $F_{(10, 497)} = 14.09^{**}$, $R^2 = .205$	
	β (*SE*)	95% CI	*β* (*SE*)	95% CI	*β* (*SE*)	95% CI
Group dominance	-.166 (*1.013*)**	[-4.98; -1.00]	-.001 (*.995*)	[-1.97; 1.94]	.184 (*.861*)**	[1.28; 4.67]
Anti-egalitarianism	.164 (*1.107*)**	[1.01; 5.37]	.083 (*1.088*)	[-.62; 3.66]	-.096 (*.941*)†	[-3.52; .18]
System justification	.056 (*1.004*)	[-1.00; 2.94]	.172 (*.986*)**	[.89; 4.77]	.120 (*.853*)*	[.18; 3.53]
Political orientation	.023 (*.923*)	[-1.35; 2.28]	.055 (*.906*)	[-.74; 2.82]	.032 (*.784*)	[-.96; 2.12]
Tolerance to inequality	-.165 (*1.206*)**	[-5.97; -1.23]	-.146 (*1.185*)*	[-5.35; -.69]	.030 (*1.025*)	[-1.43; 2.60]
Hostile classism	-.267 (*.937*)**	[-6.32; -2.63]	-.074 (*.920*)	[-2.99; .63]	.219 (*.796*)**	[1.73; 4.86]
Protective paternalist	.022 (*.657*)	[-.99; 1.59]	.120 (*.645*)*	[.32; 2.86]	.102 (*.558*)*	[.19; 2.39]
Complementary class differentiation	.046 (*.763*)	[-.70; 2.30]	-.061 (*.750*)	[-2.47; .47]	-.115 (*.649*)**	[-3.08; -.53]
Subjective social class	.092 (*.726*)*	[.00; 2.86]	.121 (*.713*)*	[.38; 3.18]	.025 (*.617*)	[-.86; 1.56]
Objective social class	-.070 (*.349*)	[-1.21; .16]	-.009 (*.343*)	[-.74; .61]	.069 (*.297*)	[-.12; 1.04]

Note. SES = Socioeconomic status; ** $p \leq .001$; * $p \leq .05$; † $p \leq .077$.

Table SM-7. *Multiple Regression Analysis of Ideological Variables on the Attributions about Poverty and Wealth in Study 1.*

	Internal attributions about poverty $F_{(10, 495)} = 49.94^{**}$, $R^2 = .492$		External attributions about poverty $F_{(10, 495)} = 20.59^{**}$, $R^2 = .279$		Internal attributions about wealth $F_{(10, 492)} = 44.62^{**}$, $R^2 = .465$		External attributions about wealth $F_{(10, 492)} = 10.50^{**}$, $R^2 = .159$	
	β (*SE*)	95% CI	*β* (*SE*)	95% CI	*β* (*SE*)	95% CI	*β* (*SE*)	95% CI
Group dominance	-.005 (*.051*)	[-.11; .10]	-.070 (*.044*)	[-.15; .03]	.121 (*.048*)**	[.04; .23]	.018 (*.041*)	[-.07; .10]
Anti-egalitarianism	.044 (*.056*)	[-.05; .17]	-.184 (*.049*)**	[-.27; -.08]	-.047 (*.052*)	[-.16; .05]	-.135 (*.045*)*	[-.20; -.02]
System justification	.385 (*.051*)**	[.35; .55]	-.177 (*.044*)**	[-.24; -.06]	.505 (*.048*)**	[.44; .62]	-.251 (*.041*)**	[-.26; -.10]
Political orientation	.066 (*.047*)†	[.00; .18]	-.008 (*.040*)	[-.09; .07]	.126 (*.044*)**	[.07; .24]	-.049 (*.038*)	[-.12; .03]
Tolerance to inequality	.017 (*.061*)	[-.10; .15]	-.212 (*.053*)**	[-.33; -.12]	.005 (*.057*)	[-.11; .12]	-.150 (*.049*)**	[-.23; -.04]
Hostile classism	.344 (*.047*)**	[.29; .48]	.014 (*.041*)	[-.07; .09]	-.001 (*.044*)	[-.09; .09]	.123 (*.038*)*	[.01; .16]
Protective paternalist	.032 (*.033*)	[-.04; .10]	.026 (*.029*)	[-.04; .08]	.171 (*.031*)**	[.09; .21]	.031 (*.027*)	[-.04; .07]
Complementary class differentiation	-.011 (*.039*)	[-.09; .06]	.166 (*.033*)**	[.08; .21]	.034 (*.036*)	[-.04; .11]	.146 (*.031*)**	[.04; .17]
Subjective social class	-.001 (*.037*)	[-.07; .07]	-.022 (*.029*)	[-.07; .04]	.039 (*.034*)	[-.03; .11]	-.021 (*.030*)	[-.07; .05]
Objective social class	.023 (*.018*)	[-.02; .05]	-.028 (*.000*)	[.00; .00]	.017 (*.017*)	[-.03; .04]	.024 (*.014*)	[-.02; .04]

Note. ** $p \leq .001$; * $p \leq .05$; † $p \leq .058$.

Table SM-8. *Multiple Regression Analysis of Ideological Variables on the Blaming Low- and Praising High-SES groups variables in Study 1.*

	Blaming Low-SES groups $F_{(10, 495)} = 64.96^{**}$, $R^2 = .559$		Praising High-SES groups $F_{(10, 492)} = 50.92^{**}$, $R^2 = .499$	
	β (*SE*)	95% CI	β (*SE*)	95% CI
Group dominance	.016 (*.056*)	[-.09; .13]	.095 (*.065*)*	[.02; .27]
Anti-egalitarianism	.128 (*.062*)**	[.08; .32]	.028 (*.071*)	[-.09; .19]
System justification	.427 (*.056*)**	[.47; .69]	.516 (*.064*)**	[.63; .88]
Political orientation	.056 (*.051*)†	[-.01; .19]	.148 (*.059*)**	[.14; .37]
Tolerance to inequality	.102 (*.067*)**	[.04; .31]	.083 (*.077*)*	[.00; .31]
Hostile classism	.233 (*.052*)**	[.21; .41]	-.058 (*.060*)	[-.20; .04]
Protective paternalist	.006 (*.037*)	[-.07; .08]	.130 (*.042*)**	[.07; .24]
Complementary class differentiation	-.092 (*.043*)**	[-.21; -.04]	-.050 (*.049*)	[-.17; .02]
Subjective social class	-.004 (*.040*)	[-.08; .07]	.049 (*.046*)	[-.03; .16]
Objective social class	.031 (*.019*)	[-.02; .06]	.000 (*.022*)	[-.04; .04]

Note. SES = Socioeconomic status; ** $p \leq .001$; * $p \leq .05$; † $p \leq .085$.

Table SM-9. *Multiple Regression Analysis of Ideological Variables on the Support for Social Policies in Study 1.*

	Redistribution policies $F_{(10, 489)} = 35.44^{**}$, $R^2 = .408$		Progressive taxation $F_{(10, 489)} = 17.531^{**}$, $R^2 = .249$		Welfare policies $F_{(10, 489)} = 17.00^{**}$, $R^2 = .243$	
	β (*SE*)	95% CI	β (*SE*)	95% CI	β (*SE*)	95% CI
Group dominance	-.180 (*.056*)**	[-.33; -.11]	-.037 (*.071*)	[-.19; .09]	-.028 (*.055*)	[-.14; .08]
Anti-egalitarianism	-.168 (*.062*)**	[-.34; -.10]	-.066 (*.078*)	[-.25; .06]	-.161 (*.061*)**	[-.30; -.06]
System justification	-.198 (*.056*)**	[-.34; -.12]	-.109 (*.071*)*	[-.28; .00]	-.126 (*.055*)*	[-.24; -.02]
Political orientation	-.117 (*.052*)**	[-.26; -.06]	-.268 (*.066*)**	[-.54; -.28]	-.116 (*.051*)**	[-.24; -.04]
Tolerance to inequality	-.182 (*.067*)**	[-.40; -.14]	-.111 (*.085*)*	[-.35; -.02]	-.144 (*.066*)**	[-.32; -.06]
Hostile classism	-.011 (*.053*)	[-.12; .09]	-.115 (*.066*)*	[-.28; -.02]	-.132 (*.052*)*	[-.23; -.03]
Protective paternalist	-.052 (*.037*)	[-.12; .02]	-.010 (*.046*)	[-.10; .08]	.031 (*.036*)	[-.05; .10]
Complementary class differentiation	.054 (*.042*)	[-.02; .15]	.063 (*.054*)	[-.02; .19]	.090 (*.042*)*	[.01; .18]
Subjective social class	-.008 (*.037*)	[-.08; .07]	-.028 (*.051*)	[-.13; .07]	.055 (*.040*)	[-.03; .13]
Objective social class	-.044 (*.000*)	[.00; .00]	-.020 (*.025*)	[-.06; .04]	-.006 (*.019*)	[-.04; .04]

Note. SES = Socioeconomic status; ** $p \le .001$; * $p \le .05$.

Table SM-10. *Multiple Regression Analysis of the Attribution of Humanity to Low- and High-SES groups on the Attributions about Poverty and Wealth in Study 1.*

	Internal attributions about poverty $F_{(4, 501)} = 16.30^{**}$, $R^2 = .108$		External attributions about poverty $F_{(4, 501)} = 6.93^{**}$, $R^2 = .045$		Internal attributions about wealth $F_{(4, 498)} = 21.26^{**}$, $R^2 = .139$		External attributions about wealth $F_{(4, 498)} = 2.05^{\dagger}$, $R^2 = .008$	
	β (SE)	95% CI	*β (SE)*	95% CI	*β (SE)*	95% CI	*β (SE)*	95% CI
Low-SES Humanity	-.370 (*.003*)**	[-.03; -.02]	.265 (*.003*)**	[.01; .02]	-.356 (*.003*)**	[-.03; -.02]	.084 (*.002*)	[.00; .01]
High-SES Humanity	.289 (*.004*)**	[.01; .03]	-.167 (*.003*)**	[-.01; .00]	.388 (*.003*)**	[.02; .03]	-.133 (*.002*)*	[-.01; .00]
SSC	.127 (*.048*)**	[.04; .23]	-.070 (*.036*)	[-.12; .02]	.149 (*.043*)**	[.06; .22]	-.055 (*.032*)	[-.10; .03]
OSC	-.090 (*.023*)†	[-.09; .00]	.038 (*.017*)	[-.02; .05]	-.072 (*.021*)	[-.07; .01]	.053 (*.015*)	[-.01; .05]

Note. SES = Socioeconomic status; SSC = Subjective social class; OSC = Objective social class; ** $p \leq .001$; * $p \leq .05$; † $p \leq .085$.

Table SM-11. *Multiple Regression Analysis of Humanity Attributions on the Blaming Low- and Praising High-SES groups variables in Study 1.*

	Blaming Low-SES groups $F_{(4, 501)} = 18.04^{**}$, $R^2 = .119$		Praising High-SES groups $F_{(4, 498)} = 21.64^{**}$, $R^2 = .141$	
	β (SE)	95% CI	*β (SE)*	95% CI
Low-SES Humanity	-.395 (*.004*)**	[-.04; -.02]	-.356 (*.004*)**	[-.04; -.02]
High-SES Humanity	.302 (*.004*)**	[.02; .03]	.390 (*.005*)**	[.03; .04]
SSC	.120 (*.056*)**	[.04; .26]	.154 (*.060*)**	[.09; .32]
OSC	-.078 (*.027*)†	[-.10; .01]	-.087 (*.029*)†	[-.11; .00]

Note. SES = Socioeconomic status; SSC = Subjective social class; OSC = Objective social class; ** $p \leq .001$; * $p \leq .05$; † $p \leq .086$.

Table SM-12. *Multiple Regression Analysis of the Attributions of Humanity to Low- and High-SES groups on the Support for Social Policies in Study 1.*

	Redistribution policies $F_{(4, 495)} = 10.25^{**}$, $R^2 = .069$		Progressive taxation $F_{(4, 495)} = 11.24^{**}$, $R^2 = .076$		Welfare policies $F_{(4, 495)} = 1.68$, $R^2 = .005$	
	β (*SE*)	95% CI	β (*SE*)	95% CI	β (*SE*)	95% CI
Low-SES Humanity	.295 (*.004*)**	[.01; .03]	.307 (*.004*)**	[.02; .03]	.132 (*.003*)*	[.00; .01]
High-SES Humanity	-.266 (*.004*)**	[-.03; -.01]	-.274 (*.004*)**	[-.03; -.01]	-.071 (*.003*)	[-.01; .00]
SSC	-.085 (*.050*)†	[-.19; .01]	-.094 (*.056*)*	[-.22; .00]	-.021 (*.045*)	[-.11; .07]
OSC	.048 (*.024*)	[-.02; .07]	.046 (*.027*)	[-.03; .08]	.052 (*.022*)	[-.02; .07]

Note. SES = Socioeconomic status; SSC = Subjective social class; OSC = Objective social class; ** $p \leq .001$; * $p \leq .05$; † $p \leq .085$.

Table SM-13. *Multiple Regression Analysis of the Attributions about Poverty and Wealth on the Support for Social Policies in Study 1.*

	Redistribution policies $F_{(6, 493)} = 40.81^{**}$, $R^2 = .324$		Progressive taxation $F_{(6, 493)} = 25.72^{**}$, $R^2 = .229$		Welfare policies $F_{(6, 493)} = 22.44^{**}$, $R^2 = .205$	
	β (*SE*)	95% CI	β (*SE*)	95% CI	β (*SE*)	95% CI
Internal attributions about poverty	-.137 (*.047*)**	[-.23; -.05]	-.163 (*.056*)**	[-.29; -.08]	-.215 (*.044*)**	[-.28; -.10]
External attributions about poverty	.268 (*.061*)**	[.26; .49]	.176 (*.072*)**	[.13; .42]	.307 (*.057*)**	[.26; .48]
Internal attributions about wealth	-.302 (*.052*)**	[-.44; -.24]	-.244 (*.062*)**	[-.43; -.18]	-.086 (*.049*)†	[-.18; .01]
External attributions about wealth	.092 (*.070*)*	[.01; .29]	.091 (*.084*)*	[.00; .33]	.025 (*.066*)	[-.10; .17]
SSC	-.004 (*.043*)	[-.09; .08]	-.028 (*.051*)	[-.13; .07]	.049 (*.040*)	[-.03; .12]
OSC	-.002 (*.021*)	[-.04; .04]	.000 (*.025*)	[-.05; .05]	.017 (*.019*)	[-.03; .05]

Note. SSC = Subjective social class; OSC = Objective social class; ** $p \leq .001$; * $p \leq .05$; † $p \leq .089$.

Table SM-14. *Multiple Regression Analysis of Blaming Low- and Praising High-SES groups variables on the Support for Social Policies in Study 1.*

	Redistribution policies $F_{(4, 495)} = 63.61^{**}$, $R^2 = .334$		Progressive taxation $F_{(4, 495)} = 43.45^{**}$, $R^2 = .254$		Welfare policies $F_{(4, 495)} = 29.44^{**}$, $R^2 = .186$	
	β (*SE*)	95% CI	β (*SE*)	95% CI	β (*SE*)	95% CI
Blaming Low-SES	-.239 (*.042*)**	[-.29; -.12]	-.184 (*.050*)**	[-.28; -.08]	-.352 (*.040*)**	[-.34; -.18]
Praising High-SES	-.397 (*.039*)**	[-.40; -.24]	-.365 (*.047*)**	[-.42; -.24]	-.121 (*.038*)*	[-.16; -.01]
SSC	.001 (*.043*)	[-.08; .09]	-.022 (*.050*)	[-.13; .07]	.048 (*.041*)	[-.04; .12]
OSC	-.007 (*.020*)	[-.04; .04]	-.003 (*.024*)	[-.05; .05]	.016 (*.020*)	[-.03; .05]

Note. SES = Socioeconomic status; SSC = Subjective social class; OSC = Objective social class; ** $p \leq .001$; * $p \leq .05$

D) Supplementary details and analysis of the Studies 2a to 2c.

In the following pages we materials used in the Studies 2a to 2c to manipulate the ideological variables. After agreeing to participate participants were presented with the information of the fictitious society (Figure SM-2) and then asked to read a short description of the way of thinking they were assigned to (Figures SM-2 to SM-5).

"Una nueva vida en Bimbola"

Imagína que te vas a vivir a un nuevo país llamado Bimbola.

Vas a comenzar una nueva vida allí y te convertirás en un nuevo miembro de la sociedad bimboleana.

Bimbola es como cualquier otra sociedad. Similar al del país donde vives y su cultura y forma de vida es muy similar a la cultura y forma de ser de México.
En lo que respecta a su estructura socioeconómica:

- Bimbola tiene su propia moneda, el **Dólar Bimboleano (DB)**. Esta moneda no tiene nada que ver con las monedas existentes en otros países (como, por ejemplo, el Peso Mexicano).
- En términos de **distribución de la riqueza**, la población de Bimbola difiere en sus ganacias mensuales, con algunos grupos que ganan más DB por año que otros. Esto significa que en Bimbola, como en otros países, parte de la población es **rica** y otra parte de la población es **pobre**.

En el siguiente cuestionario queremos que te imagines que al entrar a formar parte de esta nueva sociedad tu **forma de entender el mundo** (la forma en la que piensas, la forma en la que te relacionas con los demás o la forma en la que entiendes la sociedad) que te rodea **va a ser diferente**.

Independientemente de cómo pienses en la vida real, en las siguientes preguntas **queremos que te imagines que tu forma de entender el mundo en esta nueva sociedad va a ser como en la siguiente descripción**:

Figure SM-2. Information about the fictitious society that participants read at the beginning of the experiment (Studies 2a to 2c). *

*Translation of the information: "*A new life in Bimbola*"

Imagine you are going to live in a new country called Bimbola.

You are going to start a new life there and become a member of the Bimbolean society.

Bimbola is like any other society. Their culture and way of life is very similar to the Mexican culture and way of life. Regarding its socio-economic structure:

- Bimbola has its own currency, the **Bimbola Dollar (DB)**. This currency has nothing to do with the currencies existing in other countries (such as, for example, the Mexican Peso).
- In terms of **wealth distribution**, the population of Bimbola differs in their monthly earnings, with some groups earning more DB per year than others. This means that in Bimbola, as in other countries, part of the population is **rich** and part of the population is **poor**.

In the following quiz, **you are asked to imagine that you are a citizen of Bimbola**. Like all citizens, you will be part of a certain social group, which has certain ideas and perspectives on society. **Below we are going to describe how you and the group you belong to in Bimbola think,** and it is necessary that you answer this questionnaire from this point of view. Not thinking with your own ideas and beliefs, but from the point of view we are describing.

Social dominance

TU FORMA DE PENSAR Y LA DEL GRUPO AL QUE PERTENECES EN BIMBOLA SERÁ LA SIGUIENTE.
POR FAVOR LEE ATENTAMENTE ESTA INFORMACIÓN:

La gente como yo, considera que en la sociedad en la que vivimos **algunos grupos de personas son simplemente inferiores** a otros grupos de personas. Por esta razón, los grupos que están debajo de los demás en la sociedad no merecen recibir los mismos recursos, beneficios o derechos que los grupos de personas que están arriba o en la parte superior de la jerarquía social. La gente como yo también piensa que para que la sociedad funcione, **algunos grupos de personas tienen que estar arriba**, tienen que tener más poder, y otros tienen que estar abajo. Esto refleja el orden natural de las cosas, en el que los **grupos más aventajados y superiores deben dominar la sociedad**.

Nonsocial dominance

TU FORMA DE PENSAR Y LA DEL GRUPO AL QUE PERTENECES EN BIMBOLA SERÁ LA SIGUIENTE.
POR FAVOR LEE ATENTAMENTE ESTA INFORMACIÓN:

La gente como yo, considera que en la sociedad en la que vivimos **ningún grupo de personas debería ser considerado inferior** comparado con otros grupos de personas. Por esta razón, los grupos que están debajo de los demás en la sociedad merecen recibir los **mismos recursos, beneficios o derechos** que los grupos de personas que están arriba o en la parte superior de la jerarquía social. La gente como yo también piensa que para que la sociedad funcione **ningún grupo de personas tienen que estar por encima o tener más poder que otros grupos de personas**. Esto refleja el orden natural en el que ningún grupo por muy aventajado y superior que sea debe dominar en la sociedad.

Figure SM-3. Text implemented to manipulate the social dominance vs nonsocial dominance in the study 2a.*

*Translation of the information:

Social dominance: People like me believe that, in the society in which we live, some groups of people are simply inferior to other groups of people. For this reason, groups that are below others in society do not deserve to receive the same resources, benefits or rights as groups of people who are above or at the top of the social hierarchy. People like me also think that for society to function, some groups of people must be at the top, must have more power, and others must be at the bottom. This reflects the natural order of things, in which the more advantaged and superior groups must dominate society.

Nonsocial dominance: People like me believe that, in the society in which we live, no group of people should be considered inferior compared to other groups of people. For this reason, groups that are below others in society deserve to receive the same resources, benefits or rights as groups of people who are above or at the top of the social hierarchy. People like me also think that for society to function no group of people must be above or have more power than other groups of people. This reflects the natural order in which no group no matter how advantaged and superior should dominate in society.

System justification

TU FORMA DE PENSAR Y LA DEL GRUPO AL QUE PERTENECES EN BIMBOLA SERÁ LA SIGUIENTE.
POR FAVOR LEE ATENTAMENTE ESTA INFORMACIÓN:

La gente como yo considera que en la sociedad en la que vivimos **si la gente trabaja duro, consigue casi siempre lo que quiere**. Es decir, si la gente se esfuerza lo suficiente, puede triunfar en la vida y mejorar su posición económica. Por esta razón, las personas que no progresan en la vida no deberían culpar al sistema ya que ellas mismas son las únicas culpables de su situación. La gente como yo también piensa que la mayoría de la gente que tiene una **posición económica acomodada es debido a sus propios logros** y a un sistema económico que premia a los que de verdad se esfuerzan.

Nonsystem justification

TU FORMA DE PENSAR Y LA DEL GRUPO AL QUE PERTENECES EN BIMBOLA SERÁ LA SIGUIENTE.
POR FAVOR LEE ATENTAMENTE ESTA INFORMACIÓN:

La gente como yo considera que en la sociedad en la que vivimos **aunque la gente trabaje duro, no siempre consigue lo que quiere**. Es decir, aunque la gente se esfuerce lo suficiente puede que no triunfen en su vida y no mejoren su posición económica. Por esta razón, las personas que no progresan en la vida no deberían culparse a sí mismas ya que el sistema tiene mucha responsabilidad en esta situación. La gente como yo también piensa que hay mucha gente que tiene una **posición económica desaventajada que es el resultado de las barreras externas** y de un sistema económico que no siempre premia a los que de verdad se esfuerzan.

Figure SM-4. Text implemented to manipulate the system justification vs nonsystem justification in the Study 2b.*

*Translation of the information:

System justification: People like me believe that, in the society we live in, if people work hard, they almost always get what they want. That is, if people work hard enough, they can succeed in life and improve their economic position. For this reason, people who do not progress in life should not blame the system since they themselves are the only ones to blame for their situation. People like me also think that most people who are wealthy are due to their own achievements and an economic system that rewards hard-working people.

Nonsystem justification: People like me believe that, in the society in which we live, although people work hard, they don't always accomplish what they want. In other words, even though they make a considerable amount of effort, they may still not succeed in their lives nor improve their economic status. Thus, those who do not succeed in life should not blame themselves given that much of the responsibility for these cases lies on the system itself. People like me also believe that many people have a disadvantaged economic situation, which is the result of external barriers and an economic system that doesn't always reward those who make real effort.

Hostile classism

TU FORMA DE PENSAR Y LA DEL GRUPO AL QUE PERTENECES EN BIMBOLA SERÁ LA SIGUIENTE.
POR FAVOR LEE ATENTAMENTE ESTA INFORMACIÓN:

La gente como yo considera que en la sociedad en la que vivimos **las personas pobres no contribuyen en nada**. Por lo general las personas pobres **carecen de la iniciativa necesaria para salir adelante**, no saben tomar decisiones importantes en su vida por sí mismas y eso hace que acaben malgastando su dinero en drogas o alcohol. Además, la gente como yo también piensa que cuando las organizaciones benéficas o las instituciones intentan ayudarles, **las personas pobres intentan aprovecharse** y manipularlos para sacarles provecho y recibir más recursos siendo unos vagos.

Nonhostile classism

TU FORMA DE PENSAR Y LA DEL GRUPO AL QUE PERTECENES EN BIMBOLA SERÁ LA SIGUIENTE.
POR FAVOR LEE ATENTAMENTE ESTA INFORMACIÓN:

La gente como yo considera que en la sociedad en la que vivimos **las personas pobres pueden contribuir en la misma medida que el resto de personas**. Por lo general l**as personas pobres tienen iniciativa para salir adelante**, saben tomar decisiones importantes en su vida por sí mismas y eso hace que sepan administrar correctamente el poco dinero que tienen. Además, la gente como yo también piensa que cuando las organizaciones benéficas o las instituciones intentan ayudarles, las **personas pobres suelen aprovechar esos recursos** y sacarles partido para poder salir adelante en la vida.

Figure SM-5. Text implemented to manipulate the hostile classism vs nonhostile classism in the Study 2c.*

*Translation of the information:

Hostile classism: People like me believe that, in the society in which we live, poor people don’t contribute in any way. Generally, poor people lack the necessary initiative to move

ahead; they do not know how to make important life decisions by themselves and this leads them to waste away their money on alcohol and drugs. Furthermore, people like me believe that when charity organizations or institutions try to help them, poor people try to take advantage and manipulate them to exploit them and receive more resources as they are lazy people.

Nonhostile classism: People like me believe that, in the society in which we live, poor people can contribute in the same manner as the rest of the population. In general, poor people have the initiative to move ahead and they know how to take important decisions by themselves, which makes them able to effectively administer the small amount of money they possess. Furthermore, people like me believe that when charity organizations or institutions try to help them, poor people usually make good use of these resources and employ them wisely to move forward in life.

Alternative ANCOVA analysis

In the studies 2a to 2c participants were additionally asked to report, at the end of the survey, to what extent they believe that their personal ideological point of view was according to or not to the ideology they were assigned to in Bimbola (i.e., experimental condition). Answers were from 1 (Completely different) to 7 (Completely equal). We compute alternative ANCOVA analysis controlled by this variable in the Studies 2a to 2c (Table SM-17). Results, in general, indicate that even when this variable exerted certain influence on the results, the experimental effect of the manipulation was significant above and beyond this covariate. Thus, confirming the effectiveness of our manipulation and its effect on the variables we are studying.

Table SM-15. *Alternative ANCOVA analysis controlled by the Participants Perceived Similarity of their Ideological Point of View with the Ideological Point of View they were Assigned to in Bimbola for the Studies 2a to 2c.*

	Humanity gap	Blaming low-SES groups	Praising high-SES groups	Welfare policies	Redistribution policies	Progressive taxation
Main effect 3a	$F_{(1, 205)}$ = 206.91, p < .001, η^2_p= .505	$F_{(1, 205)}$ = 139.75, p < .001, η^2_p= .408	$F_{(1, 205)}$ = 27.29, p < .001, η^2_p= .118	$F_{(1, 205)}$ = 97.82, p < .001, η^2_p= .325	$F_{(1, 205)}$ = 172.85, p < .001, η^2_p= .460	$F_{(1, 205)}$ = 78.76, p < .001, η^2_p= .280
Ideological similarity of the participants 3a	$F_{(1, 205)}$ = 13.34, p < .001, η^2_p= .062	$F_{(1, 205)}$ = 33.45, p < .001, η^2_p= .141	$F_{(1, 205)}$ = 4.72, p = .031, η^2_p= .023	$F_{(1, 205)}$ = 27.37, p < .001, η^2_p= .119	$F_{(1, 205)}$ = 18.90, p < .001, η^2_p= .085	$F_{(1, 205)}$ = 18.02, p < .001, η^2_p= .082
Main effect 3b	$F_{(1, 211)}$ = 17.65, p < .001, η^2_p= .078	$F_{(1, 211)}$ = 209.45, p < .001, η^2_p= .501	$F_{(1, 211)}$ = 230.69, p < .001, η^2_p= .525	$F_{(1, 211)}$ = 126.20, p < .001, η^2_p= .376	$F_{(1, 211)}$ = 178.02, p < .001, η^2_p= .460	$F_{(1, 211)}$ = 126.47, p < .001, η^2_p= .377
Ideological similarity of the participants 3b	$F_{(1, 211)}$ = 21.44, p < .001, η^2_p= .093	$F_{(1, 211)}$ = 25.81, p < .001, η^2_p= .110	$F_{(1, 211)}$ = 7.36, p = .007, η^2_p= .034	$F_{(1, 211)}$ = 11.10, p = .001, η^2_p= .050	$F_{(1, 211)}$ = 9.65, p = .002, η^2_p= .044	$F_{(1, 211)}$ = 11.79, p = .001, η^2_p= .053
Main effect 3c	$F_{(1, 212)}$ = 92.60, p < .001, η^2_p= .306	$F_{(1, 212)}$ = 108.08, p < .001, η^2_p= .340	$F_{(1, 212)}$ = 22.74, p < .001, η^2_p= .098	$F_{(1, 212)}$ = 49.80, p < .001, η^2_p= .192	$F_{(1, 212)}$ = 35.61, p < .001, η^2_p= .145	$F_{(1, 212)}$ = 58.85, p < .001, η^2_p= .219
Ideological similarity of the participants 3c	$F_{(1, 212)}$ = 59.58, p < .001, η^2_p= .221	$F_{(1, 212)}$ = 116.43, p < .001, η^2_p= .357	$F_{(1, 212)}$ = 71.43, p < .001, η^2_p= .254	$F_{(1, 212)}$ = 69.72, p < .001, η^2_p= .249	$F_{(1, 212)}$ = 45.88, p < .001, η^2_p= .179	$F_{(1, 212)}$ = 65.01, p < .001, η^2_p= .236

Complementary information of the sequential mediational analyses for Study 1 to Studies 2a-2c

In the following pages you will find the full information of the mediational analysis with information of the different effects of the models in different tables. This material was excluded from the main text due to space limitations on it.

Table SM-16. *Sequential Mediational Analysis of Humanity Gap (Mediator 1) and Blaming Low-SES Groups (Mediator 2) in Relationship between Ideological Variables and Support for Social Change Policies (Study 1)*

	Welfare Policies		Redistribution Policies		Progressive taxation	
	IE (*SE*)	95% *CI*	IE (*SE*)	95% *CI*	IE (*SE*)	95% *CI*
Total effect						
Group Dominance	-.358 *(.045)*	[-.447, -.270]	-.603 *(.047)*	[-.696, -.510]	-.413 *(.058)*	[-.528, -.299]
System Justification	-.383 *(.043)*	[-.469, -.298]	-.583 *(.047)*	[-.674, -.492]	-.483 *(.056)*	[-.592, -.374]
Hostile Classism	-.339 *(.042)*	[-.423, -.256]	-.439 *(.048)*	[-.533, -.345]	-.421 *(.054)*	[-.528, -.314]
Direct effect						
Group Dominance	-.209 *(.049)*	[-.306, -.114]	-.401 *(.047)*	[-.501, -.299]	-.151 *(.063)*	[-.275, -.028]
System Justification	-.168 *(.057)*	[-.280, -.056]	-.324 *(.061)*	[-.445, -.204]	-.163 *(.072)*	[-.306, -.019]
Hostile Classism	-.167 *(.051)*	[-.266, -.067]	-.144 *(.056)*	[-.253, -.035]	-.122 *(.065)*	[-.249, .006]
Standardized sequential indirect effect						
Group Dominance – Humanity gap – Blaming Low-SES	-.023 *(.008)*	[-.042, -.009]	-.020 *(.008)*	[-.038, -.007]	-.020 *(.007)*	[-.037, -.008]
System Justification – Humanity gap – Blaming Low-SES	-.013 *(.006)*	[-.027, -.003]	-.011 *(.006)*	[-.024, -.002]	-.011 *(.006)*	[-.024, -.003]
Hostile Classism – Humanity gap – Blaming Low-SES	-.012 *(.006)*	[-.026, -.002]	-.019 *(.009)*	[-.038, -.003]	-.015 *(.007)*	[-.031, -.002]

Note. SES = Socioeconomic status; coefficients are standardized; participants' objective and subjective socioeconomic status were included as covariates.

Table SM-17. *Sequential Mediational Analysis of Humanity Gap (Mediator 1) and Praising High-SES groups (Mediator 2) in Relationship between Ideological Variables and Support for Social Change Policies (Study 1)*

	Welfare Policies		Redistribution Policies		Progressive taxation	
	IE (*SE*)	95% *CI*	IE (*SE*)	IE (*SE*)	IE (*SE*)	95% *CI*
Total effect						
Group Dominance	-.358 *(.045)*	[-.447, -.270]	-.603 *(.047)*	[-.696, -.510]	-.413 *(.058)*	[-.528, -.299]
System Justification	-.383 *(.043)*	[-.469, -.298]	-.583 *(.047)*	[-.674, -.492]	-.483 *(.056)*	[-.592, -.374]
Hostile Classism	-.339 *(.042)*	[-.423, -.256]	-.439 *(.048)*	[-.533, -.345]	-.421 *(.054)*	[-.528, -.314]
Direct effect						
Group Dominance	-.267 *(.049)*	[-.363, -.171]	-.392 *(.048)*	[-.486, -.297]	-.144 *(.060)*	[-.261, -.027]
System Justification	-.266 *(.057)*	[-.379, -.153]	-.261 *(.058)*	[-.375, -.146]	-.085 *(.070)*	[-.222, .051]
Hostile Classism	-.269 *(.046)*	[-.359, -.177]	-.216 *(.048)*	[-.310, -.122]	-.181 *(.056)*	[-.291, -.069]
Standardized sequential indirect effect						
Group Dominance – Humanity gap – Praising High-SES	-.021 *(.006)*	[-.035, -.011]	-.031 *(.008)*	[-.048, -.018]	-.031 *(.007)*	[-.047, -.018]
System Justification – Humanity gap – Praising High-SES	-.009 *(.004)*	[-.019, -.003]	-.019 *(.005)*	[-.030, -.009]	-.019 *(.005)*	[-.032, -.009]
Hostile Classism – Humanity gap – Praising High-SES	-.024 *(.007)*	[-.039, -.013]	-.040 *(.009)*	[-.059, -.024]	-.034 *(.008)*	[-.052, -.020]

Note. SES = Socioeconomic status; coefficients are standardized; participants' objective and subjective socioeconomic status were included as covariates.

Table SM-18. *Sequential Mediational Analysis of Humanity Gap (Mediator 1) and Praising High-SES Groups (Mediator 2) on Relationship between Ideological Variables and Support for Social Change Policies (Studies 2A–2C)*

	Welfare Policies		Redistribution Policies		Progressive taxation	
	IE (*SE*)	95% *CI*	IE (*SE*)	IE (*SE*)	IE (*SE*)	95% *CI*
Total effect						
Group Dominance (vs non dominance; Study 2a)	-3.175 *(.224)*	[-3.617, -2.733]	-3.770 *(.217)*	[-4.198, -3.343]	-2.913 *(.231)*	[-3.370, -2.457]
System Justification (vs non justification; Study 2b)	-2.967 *(.204)*	[-3.369, -2.565]	-3.334 *(.200)*	[-3.729, -2.939]	-2.857 *(.201)*	[-3.254, -2.460]
Hostile Classism (vs non classism; Study 2c)	-2.410 *(.247)*	[-2.897, -1.924]	-2.237 *(.262)*	[-2.754, -1.719]	-2.425 *(.236)*	[-2.891, -1.960]
Direct effect						
Group Dominance (vs non dominance; Study 2a)	-1.036 (.324)	[-1.674, -.397]	-2.102 *(.322)*	[-2.737, -1.467]	-.981 *(.337)*	[-1.645, -.317]
System Justification (vs non justification; Study 2b)	-.734 *(.267)*	[-1.260, -.208]	-1.317 *(.274)*	[-1.857, -.777]	-.581 *(.259)*	[-1.092, -.070]
Hostile Classism (vs non classism; Study 2c)	-.466 *(.227)*	[-.914, -.018]	-.664 *(.283)*	[-1.222, -.106]	-.817 *(.236)*	[-1.282, -.352]
Standardized sequential indirect effect						
Group Dominance – Humanity gap – Praising High-SES	-.108 (.055)	[-.229, -.016]	-.118 *(.055)*	[-.237, -.024]	-.147 *(.065)*	[-.285, -.029]
System Justification – Humanity gap – Praising High-SES	-.091 *(.034)*	[-.164, -.034]	-.077 *(.029)*	[-.142, -.029]	-.096 *(.035)*	[-.174, -.037]
Hostile Classism – Humanity gap – Praising High-SES	-.247 *(.051)*	[-.358, -.158]	-.249 *(.055)*	[-.368, -.151]	-.266 *(.051)*	[-.377, -.176]

Note. SES = Socioeconomic status; coefficients are standardized; participants' objective and subjective socioeconomic status and ideologies were included as covariates.

Table SM-19. *Sequential Mediational Analysis of Humanity Gap (Mediator 1) and Blaming Low-SES Groups (Mediator 2) on Relationship between Ideological Variables and Support for Social Change Policies (Studies 2A–2C)*

	Welfare Policies		Redistribution Policies		Progressive taxation	
	IE (*SE*)	*95% CI*	IE (*SE*)	IE (*SE*)	IE (*SE*)	*95% CI*
Total effect						
Group Dominance (vs non dominance; Study 2a)	-3.175 *(.224)*	[-3.617, -2.733]	-3.770 *(.217)*	[-4.198, -3.343]	-2.913 *(.231)*	[-3.370, -2.457]
System Justification (vs non justification; Study 2b)	-2.967 *(.204)*	[-3.369, -2.565]	-3.334 *(.200)*	[-3.729, -2.939]	-2.857 *(.201)*	[-3.254, -2.460]
Hostile Classism (vs non classism; Study 2c)	-2.410 *(.247)*	[-2.897, -1.924]	-2.237 *(.262)*	[-2.754, -1.719]	-2.425 *(.236)*	[-2.891, -1.960]
Direct effect						
Group Dominance (vs non dominance; Study 2a)	-.215 *(.315)*	[-.835, .406]	-1.270 *(.315)*	[-1.891, -.648]	-.248 *(.346)*	[-.931, .435]
System Justification (vs non justification; Study 2b)	-.501 *(.245)*	[-.984, -.017]	-1.119 *(.257)*	[-1.626, -.611]	-.460 *(.246)*	[-.945, .024]
Hostile Classism (vs non classism; Study 2c)	-.070 *(.223)*	[-.370, .509]	-.079 *(.280)*	[-.632, .474]	-.303 *(.237)*	[-.771, .165]
Standardized sequential indirect effect						
Group Dominance – Humanity gap – Blaming Low-SES	-.387 *(.106)*	[-.618, -.211]	-.378 *(.100)*	[-.596, -.206]	-.399 *(.102)*	[-.629, -.230]
System Justification – Humanity gap – Blaming Low-SES	-.156 *(.041)*	[-.246, -.085]	-.131 *(.035)*	[-.208, -.071]	-.154 *(.043)*	[-.250, -.083]
Hostile Classism – Humanity gap – Blaming Low-SES	-.459 *(.074)*	[-.616, -.324]	-.487 *(.080)*	[-.659, -.347]	-.463 *(.076)*	[-.624, -.323]

Note. SES = Socioeconomic status; coefficients are standardized; participants' objective and subjective socioeconomic status and ideologies were included as covariates.
